# Decreased Expression of a Phosphoribosylanthranilate Transferase-Encoding Gene, *OsPAT1*, Causes Lesion Mimics in Rice

**DOI:** 10.3390/ijms26199428

**Published:** 2025-09-26

**Authors:** Jun Ren, Qingwen Zhang, Yafei Xu, Biaoming Zhang, Haitao Li, Yan Li, Haitao Zhang, Wenya Yuan

**Affiliations:** State Key Laboratory of Biocatalysis and Enzyme Engineering, School of Life Sciences, Hubei University, Wuhan 430062, China; renjunivy@163.com (J.R.); qingwenzyc@163.com (Q.Z.); xuyafeiwork@foxmail.com (Y.X.); zbm@hubu.edu.cn (B.Z.); lht@hubu.edu.cn (H.L.); liyan@hubu.edu.cn (Y.L.)

**Keywords:** Lesion mimic mutant, *lmm9*, *OsPAT1*, *TRP4*, tryptophan synthesis

## Abstract

Lesion mimic mutants (LMMs) represent valuable biological tools for investigating plant defense mechanisms and cell death. Although multiple genes triggering lesion mimic formation have been identified, the connection between the lesion mimic phenotype and primary nutrient biosynthesis remains poorly understood. In our study, we characterized a novel rice LMM, *lmm9*, which exhibited persistent reddish-brown necrotic lesions from seedling stage to maturity, coupled with compromised agronomic traits and increased mortality rates. Map-based cloning and whole-genome sequencing identified a causal insertion in the promoter of *Os03g03450/OsPAT1*, the sole homolog of *Arabidopsis PAT1* in rice, resulting in reduced gene expression. Genetic complementation and RNAi assays confirmed that downregulation of *OsPAT1* led to lesion mimic formation in *lmm9*. *OsPAT1* could translate into two variants—the predominant OsPAT1.1 and the C-terminal variant OsPAT1.2. Structural modeling demonstrated high conservation between OsPAT1 and yeast TRP4, and OsPAT1.1 combining the plastid signal sequence of Arabidopsis PAT1 successfully complemented the *trp4* mutant in yeast. Notably, OsPAT1.1 and OsPAT1.2 showed different localization patterns, with OsPAT1.1 targeted to mitochondria and OsPAT1.2 localized to chloroplasts. Transcription analysis showed significant upregulation of tryptophan biosynthesis pathway genes in *lmm9*, consequently increasing the relative abundance of tryptophan and associated metabolites. Our findings provided further evidence that mutations in tryptophan biosynthetic genes can induce lesion mimic phenotypes in rice and would enhance the understanding of metabolic homeostasis in plant stress responses and cell death regulation.

## 1. Introduction

Plants are constantly facing various biotic and abiotic stresses throughout their lives. As a result, they have developed many strategies to overcome these issues, such as the defense system and metabolic adaptation. Taking the interaction between plants and pathogens as an example, when pathogens adhere to the plant leaves, the leaf cells surrounding the infection sites would die in a short time, known as hypersensitive response (HR), in order to limit the spread of the pathogens. HR is a type of programmed cell death (PCD) and is usually accompanied by the burst of reactive oxygen species (ROS), the induction of defense-related genes, and the accumulation of antibacterial metabolites [1,2]. Sometimes, similar process would be activated spontaneously on plant leaves, stems or other tissues, especially when plants are mutated. Such a phenomenon is known as lesion mimic, and these mutants are called lesion mimic mutants (LMMs) [3,4]. LMMs have been isolated in many plant species, including *Arabidopsis*, barley, maize, rapeseed, rice, and wheat [3,5,6,7]. Currently, LMMs have been widely used in plant defense and cell death research, demonstrating significant potential for disease control in crop production [4,8].

A number of genes responsible for LMMs have been identified, and many of them encode enzymes participating in photosynthetic pigment metabolic pathways, particularly in tetrapyrrole and chlorophyll synthesis [9,10]. The maize *Les22* mutant, carrying a *Mutator* (*Mu*) element inserted in the uroporphyrinogen decarboxylase (UROD)-encoding gene, exhibited lesion mimic phenotype [11]. In rice, genes that encoded coproporphyrinogen III oxidase, protoporphyrinogen IX oxidase, and protochlorophyllide oxidoreductase could cause lesion mimics as well if they were mutated [9]. Moreover, the genes mutated in the maize *necrotic leaf mutant* (*nec-t*) and the soybean *Glycine max lesion mimic mutant 2-1* (*Gmlmm2-1*) also encoded coproporphyrinogen III oxidases [12,13].

Also, there are other types of genes that cause LMMs. Some of them encoded members of the ubiquitin-proteasome system, such as *SPL11*, *EBR1*, and *OsCUL3a* [14,15,16]. A few genes were involved in plant defense responses, such as *NLS1*, *CslF6*, and *SDS2* [17,18,19]. Genes that participated in transcription or epigenetic regulation also could be found in the cloned genes for LMMs [10]. As a result, the causal genes of LMMs are varied.

Besides gene mutation, improper expression of certain genes also leads to lesion mimic phenotype in plants. *RKe*, an ortholog of the bacterial blight resistance gene *Xa3/Xa26*, caused significant lesion mimics in many tissues when overexpressed in rice [20]. Upregulation of *JMJ705*, a histone lysine demethylase-encoding gene, could produce lesion mimics on rice leaves [21]. And suppression of *OsEDR1* expression in rice plants also led to lesions spontaneously on their leaves [22].

Among these genes, the first rice *LMM* genes, *Sekiguchi Lesion* (*SL*), received less attention. The Sekiguchi lesion was found in a natural mutant rice cultivar, Sekiguchi-asahi, and could be formed in the absence of pathogen [23]. The *SL* gene encodes a cytochrome P450 monooxygenase that catalyzes tryptamine conversion to serotonin and was isolated decades after its discovery [24]. Interestingly, another LMM, *early lesion leaf 1* (*ell1*), was reported as the allelic mutant of *sl* [25]. Both mutants exhibited significant changes in tryptophan content and related gene expression, revealing a potential link between tryptophan metabolism and cell death [23,26]. Recently, mutation of *EARLY LEAF LESION AND SENESCENCE 1* (*ELS1*) was also reported to induce lesion mimics and cell death in rice [27]. The *ELS1* gene encodes the α-subunit of anthranilate synthase (AS), which is involved in tryptophan biosynthesis, and is also known as *ASA2* (or *OASA2*) [27]. It was found that the expression of *ASA1* (or *OASA1*), the homolog of *ELS1/ASA2*, and other tryptophan biosynthesis pathway genes was increased in *els1*, resulting in the disruption of tryptophan metabolism [27]. Both studies established a connection between lesion mimic formation and the tryptophan biosynthesis or degradation process.

Tryptophan is an essential amino acid for all organisms and can be synthesized only in microbes and plants. Its synthetic pathway mainly involves the process from chorismate to tryptophan. The first step is the combination of chorismic acid with the amino group of glutamic acid to form anthranilate, which is the rate-limiting step and is catalyzed by AS [28,29,30]. Then anthranilate receives the phosphoribosyl group from phosphoribosyl-α1-pyrophosphate (PRPP), and generate pyrophosphate (PPi) and N-(5′-phosphoribosyl)anthranilate (PRA), which are catalyzed by the enzyme called anthranilate phosphoribosyltransferase (AnPRT) or phosphoribosylanthranilate phosphoribosyltransferase (PRT). After three more steps, tryptophan is finally synthesized from indole and serine [30].

Most of the enzymes and genes involved in tryptophan synthesis are highly conserved, while the AnPRT in *E.coli* differs a little from those in other species. In *E.coli*, this enzyme is fused with the small subunit of AS, forming a bifunctional protein encoded by the *TrpD* gene [31]. In contrast, *TRP4* gene in yeast encodes a monofunctional AnPRT enzyme [32]. The homologous gene in *Arabidopsis*, named *phosphoribosylanthranilate transferase 1* (*PAT1*), also encodes a single enzyme [33]. The *Arabidopsis trp1-1* mutant, defective in *PAT1*, displayed auxotrophy phenotypes including retarded growth and could be rescued by supplementation with indole and tryptophan [33,34]. However, research on *PAT1* homologs in other plants has rarely been reported.

Tryptophan can be degraded to generate other metabolites, such as indole-3-acetic acid (IAA), serotonin, and melatonin. Although a tryptophan-independent pathway for IAA synthesis has been reported in plants, the precursor indole is still a key component in IAA synthesis [35]. Serotonin is not only a well-studied neurotransmitter in mammals, but also a common metabolite in diverse plant species. It can be generated from tryptamine, which is a catabolite of tryptophan and has been reported to participate in plant development and stress response [36]. Melatonin, the well-known animal neurohormone, is derived from serotonin and can be used as a biocide in several plants. Recent studies have shown that melatonin coordinated with many phytohormones and regulated plant innate immunity to overcome plant diseases [37].

In our present study, we demonstrated that a mutation in another tryptophan synthesis gene, *OsPAT1*, also led to lesion mimic phenotype in rice. This LMM was discovered in the follow-up work to our previous study and was designated as *lesion mimic mutant 9* (*lmm9*). Through map-based cloning and genome sequencing, a T-DNA fragment was identified in the promoter region of the *Os03g03450/OsPAT1* gene in *lmm9*. Genetic complementation and knockdown assays confirmed that reduced *OsPAT1* expression underlies the *lmm9* phenotype. *OsPAT1* encoded two isoforms, OsPAT1.1 and OsPAT1.2, which only differed in a few residues at their C termini. OsPAT1 was evolutionarily conserved and could complement the *trp4* mutant in yeast, demonstrating its functional AnPRT activity. Interestingly, OsPAT1.1 and OsPAT1.2 were localized to mitochondria and chloroplasts, respectively. And reduced *OsPAT1* expression affected the expression of several genes in the tryptophan synthesis and degradation pathway, leading to the increased accumulation of tryptophan and other metabolites. Collectively, our results provide further evidence that tryptophan synthesis pathway genes participate in lesion mimic formation in rice.

## 2. Results

### 2.1. Discovery and Phenotypic Characterization of the lmm9 Mutant

In our previous studies of *Xa3/Xa26* gene family members, several plants with obvious lesion spots on their leaves were found in a T_1_ transgenic line, which was designed to overexpress *TRKc* in rice cultivar MDJ8. The lesion mimic phenotype was not consistent with the transgenic fragment in the same T_1_ line, and no lesions appeared in other *TRKc*-overexpressing T_1_ transgenic lines. Furthermore, the segregation ratio of the lesion mimic phenotype in the T_1_ line (Normal leaf: spotted leaf = 14:6, χ^2^ = 0.238 for 3:1, < χ^2^_0.05_ = 3.841) indicated that the phenotype was possibly caused by a single recessive Mendelian locus. Therefore, it was predicted that the phenotype might be caused by a spontaneous mutation or an unknown event during the transgenic plant regeneration process. To avoid confusion between *spotted leaf* (*spl*) mutants and *SQUAMOSA PROMOTER BINDING PROTEIN-Like* (*SPL*) genes, this mutant was named as *lmm9* here, based on recent studies [9,10].

**Figure 1:**
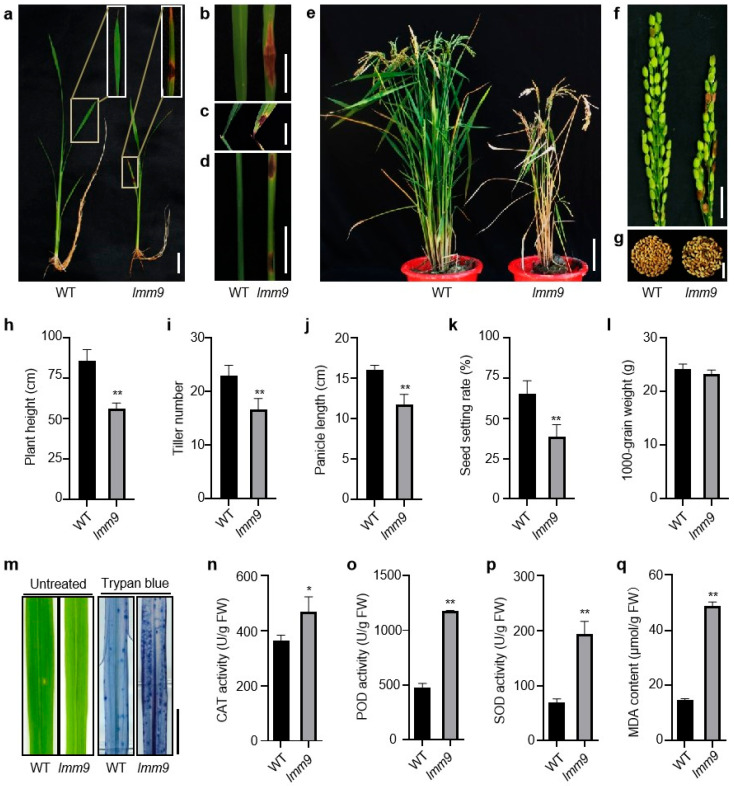
Phenotype comparison of WT and the *lmm9* mutant. (**a**) Phenotypes of WT and *lmm9* at seedling stage (about 20 d). Bar = 2 cm. (**b**–**d**) Reddish-brown spots appeared on leaf blades, collars, and leaf sheaths of *lmm9* at tillering stage. All bars = 2 cm. (**e**) Phenotypes of WT and *lmm9* at mature stage. Bar = 10 cm. (**f**,**g**) Lesions mimics appeared on the panicles and seeds in *lmm9* at mature stage. All bars = 2 cm. (**h**–**l**) Comparison of main agronomic traits of WT and *lmm9*. (**m**) Trypan blue staining of leaves of WT and *lmm9* before lesions were formed. Bar = 1 cm. (**n**–**p**) Enzyme activities of WT and *lmm9* at tillering stage. CAT, catalase; POD, peroxidase; SOD, superoxide dismutase. (**q**) Malondialdehyde (MDA) contents in the leaves of WT and *lmm9* at tillering stage. Data were presented as means ± SD of three replicates. The significance compared with WT was determined by Student’s *t*-test at *p* < 0.05 (*) and *p* < 0.01 (**).

Under natural conditions in Wuhan, reddish-brown spotted lesions appeared on the leaf blades, collars, and leaf sheaths of *lmm9* plants at as early as the seedling stage (Figure 1a–d). As the plants grew, the lesions expanded from small to large. After the lesions spread across the entire intersecting surface, the leaves would collapse and wither in several days (Figure 1e). When severe lesions developed in multiple tissues, the whole plants became shriveled. On average, more than 80 percent of the mutant plants died before harvest. Nevertheless, a few *lmm9* plants still survived to the mature stage, and lesions could be observed on peduncles, spikelets, and seeds (Figure 1e–g).

Besides the lesion mimic phenotype, the *lmm9* plants also showed differences in several agronomic traits, compared with the wildtype (WT) MDJ8. The *lmm9* plants were significantly shorter than WT in plant height (Figure 1e,h). Their tiller numbers were fewer than WT (Figure 1i). The *lmm9* plants also had shorter panicles and lower seed-setting rates (Figure 1j,k). The development of pollen, stamen, and stigma of *lmm9* was slightly affected compared with WT (Figure S1). However, no obvious difference was found in grain weight between *lmm9* and WT (Figure 1l).

Usually, cell death and ROS accumulation were closely related to lesion mimics in plants. As a result, the leaves of *lmm9* and WT were analyzed. Compared to WT, more dark blue spots appeared on *lmm9* leaves after trypan blue staining, even before lesion formation, indicating that cell death were increased in *lmm9* plants (Figure 1m). The activities of catalase (CAT), peroxidase (POD), and superoxide dismutase (SOD), which are involved in hydrogen peroxide scavenging, in *lmm9* leaves were much higher than those in WT (Figure 1n–p). The level of malondialdehyde (MDA), a metabolite of lipid peroxidation products, in *lmm9* was also higher than that in WT (Figure 1q).

Chloroplasts and mitochondria are reported to be important components for ROS and programmed cell death (PCD). To determine whether these organelles were affected in the *lmm9* plants, their ultrastructure was analyzed by transmission electron microscopy (TEM) using leaf samples. The membranes of both mitochondria and chloroplast thylakoids were very clear in the WT. However, those membranes in *lmm9* were disintegrated (Figure S2a–h). The photosynthetic pigments in leaves were also examined, and it was found that the contents of chlorophyll a, chlorophyll b, and carotenoids were all significantly reduced in *lmm9* (Figure S2i). All these results together indicated that the lesion mimics could lead to cell death and ROS accumulation in *lmm9* mutants.

### 2.2. Localization and Identification of lmm9 Gene

**Figure 2:**
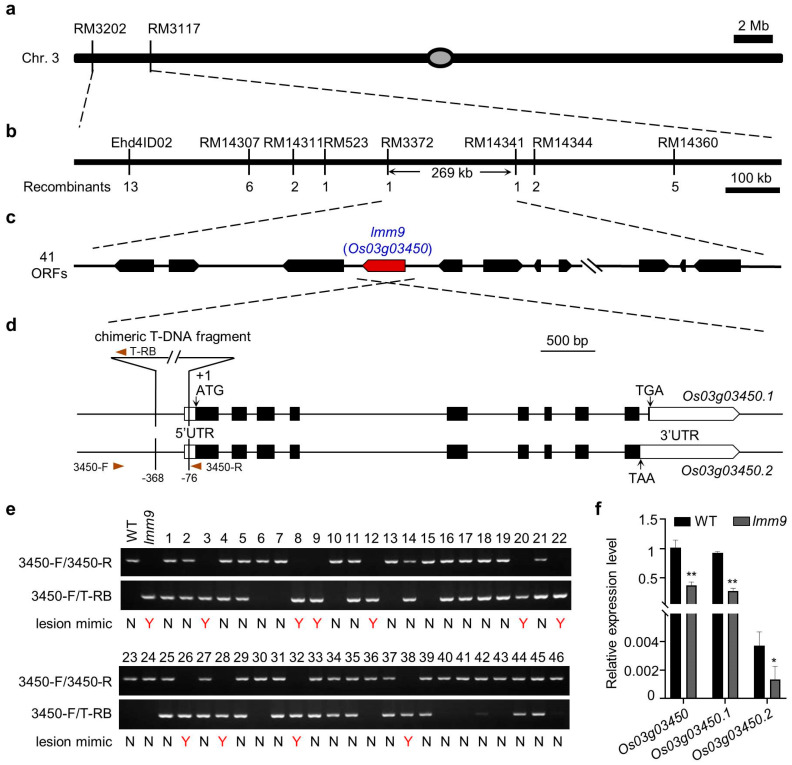
Mapping and isolation of the *lmm9* gene. (**a**) The *lmm9* gene was genetically mapped to the region between RM3202 and RM3117 on chromosome 3. (**b**) The *lmm9* gene was physically mapped to a ~269 kb region on chromosome 3. (**c**) Representative candidate ORFs in the corresponding mapping region of Nipponbare. (**d**) Schematic representation of the chimeric fragment insertion in *Os03g03450* gene and its two transcripts (*Os03g03450.1* and *Os03g03450.2*). The white boxes indicate putative UTRs. The black boxes indicate exons. The lines between the boxes indicate introns. The vertical arrows indicate the start codon and stop codon. The arrowheads indicate the positions and orientations of the PCR primers used for *LMM9* genotype analysis. (**e**) Cosegregation analysis of the offsprings of an *lmm9* heterozygous plant. All homozygous plants exhibit the lesion mimic phenotype, whereas heterozygous plants and WT do not. Y, yes; N, no. (**f**) Relative expression levels of total *Os03g03450*, *Os03g03450.1*, and *Os03g03450.2* in the leaves of WT and *lmm9* at adult stage. Data were presented as mean ± SD. The significance compared with WT was determined by Student’s *t*-test at *p* < 0.05 (*) and *p* < 0.01 (**).

To investigate which gene caused the lesion mimics phenotype, the *lmm9* mutant was used as the female parent and hybridized with MH63 (*indica*). However, no hybrids could be obtained due to the high mortality of *lmm9* mutants. Then normal plants segregated from the same lines as the *lmm9* mutants, which carried the *lmm9* gene at a rate of two-thirds, were chosen as the female parent to hybridize with MH63 again. These plants were also checked for lesion formation in their self-fertilized offspring to determine which plant carried the *lmm9* gene. Fortunately, one heterozygous plant was found, and its hybrids were self-crossed and harvested separately for generating segregating populations. Two populations were analyzed, and plants in one population exhibited the same lesion mimic phenotype. Using this population, the *lmm9* gene was mapped to a 269 kb genomic region between markers RM3372 and RM14341 on chromosome 3 (Figure 2a,b). In this region, a total of forty-one genes were found according to the reference genome of Nipponbare (Figure 2c).

At the same time, whole-genome sequencing was performed using *lmm9* and WT plants. In the mapping region, a large fragment insertion in the promoter of gene *Os03g03450* and ten minor differences in other genes were found in *lmm9* compared with WT. However, only the insertion could be validated by Sanger sequencing in the subsequent analysis. After identification of the flanking sequences on both sides, it was found that the inserted fragment was an unusual chimera of two segments that was originated from pU1301 vector (not including the *GUS* reporter gene) and the *Agrobacterium* Ti plasmid. The fragment was inserted into and replaced the sequence between 368 and 75 base pair upstream of the start codon of *Os03g03450* (Figure 2d). To confirm the relationship between the insertion and lesion mimic phenotype, a pair of primers was designed in the flanking sequences and was used to analyze the offsprings of an *lmm9* heterozygous plant. In these plants, all the lesion mimic plants were homozygous for the inserted fragment and none of the heterozygous plants developed lesions (Figure 2e). These results suggested that *Os03g03450* was probably the *lmm9* gene.

Since the insertion occurred in the promoter and untranslated region (UTR) rather than the coding region, the expression of *Os03g03450* in *lmm9* was analyzed. There were two transcripts for this gene, *Os03g03450.1* and *Os03g03450.2*, which were identical in most parts except for the 3′ terminal sequences (Figure 2d). It was found that the total expression of *Os03g03450* in the leaves of *lmm9* mutant was much lower than that in WT (Figure 2f). *Os03g03450.1* accounted for a large portion of the total transcripts, whereas both *Os03g03450.1* and *Os03g03450.2* were less abundant in *lmm9* than in WT (Figure 2f and Figure S3).

### 2.3. Downregulation of Os03g03450 Led to the lmm9 Mutant Phenotype

**Figure 3:**
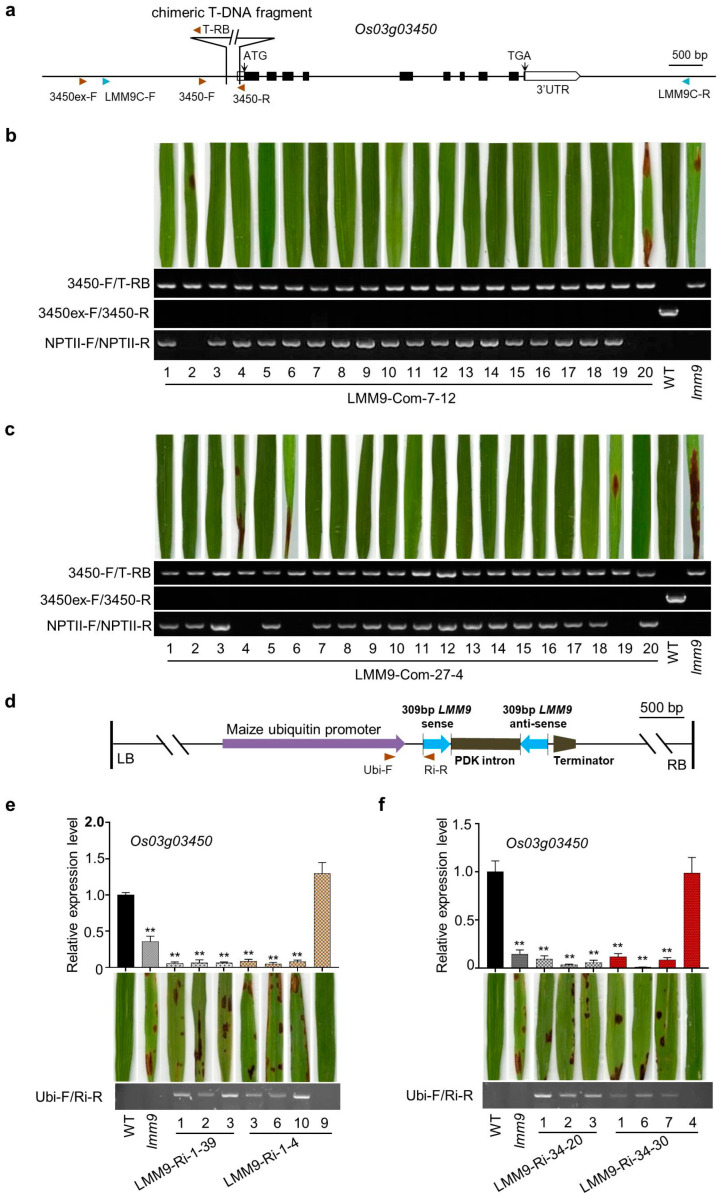
*Os03g03450* was responsible for *lmm9* mutant. (**a**) Schematic representation of the insertion in *Os03g03450* gene and primers used in the complement test. (**b**,**c**) The lesion mimic phenotype of *lmm9* could be recovered in the positive transgenic plants in LMM9-Com-7-12 (**b**) and LMM9-Com-27-4 (**c**) T_2_ generation. NPTII-F/NPTII-R, primers used for detecting the LMM9-Com transgenic plants. (**d**) Schematic representation of the vector used in LMM9-Ri plants. (**e**,**f**) The lesion mimic phenotype was observed in LMM9-Ri plants. Relative expression levels of *Os03g03450* were presented as means ± SD of three biological replicates. Significant differences compared with WT (Student’s *t*-test, *p* < 0.01) were shown as double stars (**).

To further confirm whether the *lmm9* phenotype was caused by the fragment insertion in the promoter of *Os03g03450*, a native promoter-driven *Os03g03450* fragment was transformed into the *lmm9* mutant, generating LMM9-Com plants (Figure 3a). Among the forty-nine T_0_ transgenic plants, all positive plants no longer developed lesions. Meanwhile, all negative plants remained smaller than WT and developed the same lesions as *lmm9*. The results were further confirmed in several T_1_ and T_2_ generation lines. In the positive transgenic plants of LMM9-Com-7-12 and LMM9-Com-27-4 lines, the growth was restored and no lesions were found (Figure 3b,c). However, the expression of *Os03g03450* in LMM9-Com-7 and LMM9-Com-27 was higher than that in WT, likely due to the position effect from transformation (Figure S4). These results suggested that *Os03g03450* was the *lmm9* gene.

To prove that the reduced *Os03g03450* expression was crucial for lesion formation, RNAi plants (LMM9-Ri) were generated (Figure 3d). In the T_0_ generation, 17 out of 36 plants developed significant lesion mimics similar to the *lmm9* mutant and died during growth, leaving only 19 surviving plants with fewer lesions. The lesion mimics could be observed in their T_1_ and T_2_ lines. In four different LMM9-Ri T_2_ lines, the *Os03g03450* expression levels in positive transgenic plants were significantly suppressed and lesions developed on their leaves, whereas negative plants showed normal gene expression and growth (Figure 3e,f). These results indicated that the reduced expression of *Os03g03450*, caused by the promoter insertion, was responsible for the *lmm9* mutant phenotype.

Furthermore, site-directed mutagenesis was performed to determine whether *Os03g03450* knockout could also induce lesion mimics. Two target sites were selected for CRISPR/Cas9-mediated mutation to generate LMM9-KO plants (Figure S5a). However, only the target in the second exon was mutated in these plants. During the selection for four generations, some plants were found to develop lesions resembling *lmm9*. Sequencing data revealed that these plants were chimeric mutants harboring multiple allelic mutations in *Os03g03450*. Unfortunately, these plants all died before harvest. Similarly, in the offspring of plants carrying heterozygous mutations, no homozygous mutants were found. For example, in the offspring of the heterozygous LMM9-KO-T3-10 line, all the plants were either heterozygous or wildtype at the *Os03g03450* locus, with no homozygous mutant plants identified (Figure S5b,c). Given the high fatality rate of the *lmm9* mutants, these results indicated that *Os03g03450* is essential for rice development and that its knockout is lethal.

### 2.4. Os03g03450 Encoded a Conserved and Functional Enzyme–OsPAT1

According to the gene annotation, *Os03g03450* encoded a putative AnPRT enzyme, which was homologous to *Arabidopsis* PAT1 and yeast TRP4. As a result, this gene was renamed as *OsPAT1* hereafter. In rice genome, there were no other genes homologous to *OsPAT1*, which was consistent with the high fatality of both *lmm9* and LMM9-KO plants during growth. A search for homologous proteins of OsPAT1.1 and OsPAT1.2 in other species were carried out based on the NCBI database. All the homologs could be grouped into 4 categories, which mainly corresponded to dicots, monocots, spore plants, and microbes (Figure S6). This phenomenon indicated that these proteins were conserved during evolution.

**Figure 4:**
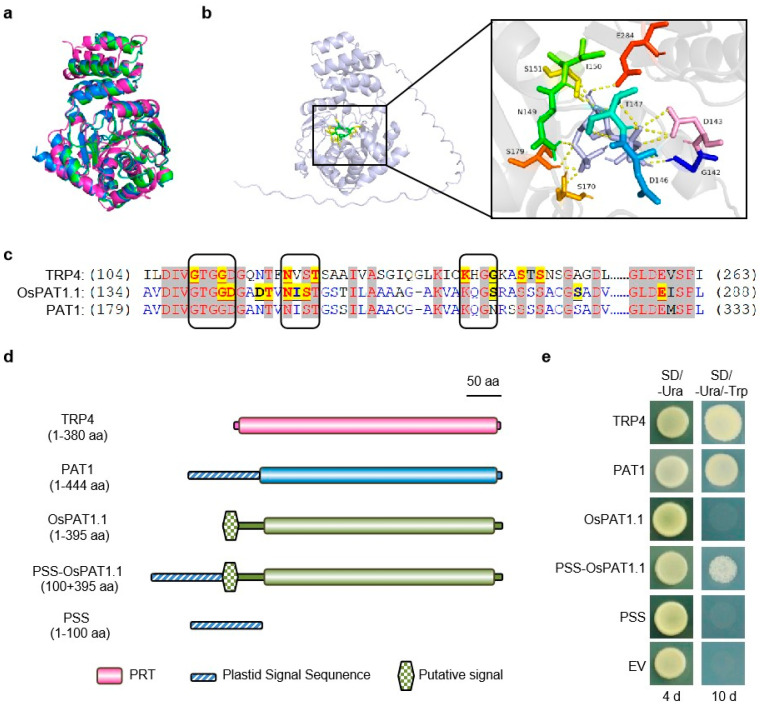
Functional analysis of OsPAT1 protein. (**a**) Three-dimensional structure comparison of PAT1 (Blue) and OsPAT1.1 (Green) predicted by AlphaFold 3 with yeast TRP4 (Magenta). (**b**) Predicted interactions between OsPAT1.1 and PRPP. PRPP was colored in gray. The PRPP-interacting residues were shown in rainbow colors. (**c**) Alignment of the PRPP-interacting regions of TRP4, PAT1, and OsPAT1.1. Boxes indicated the conserved motifs in reported AnPRTs. PRPP-interacting residues were highlighted and underlined. (**d**,**e**) Combined with the plastid signal sequence of PAT1, the modified OsPAT1.1 complemented the yeast *trp4* mutant on tryptophan-deficient medium. PSS, Plastid signal sequence; EV, empty vector.

Nonetheless, OsPAT1.1 and OsPAT1.2 exhibited only 56% sequence identity to *Arabidopsis* PAT1 (Figure S7). To determine whether OsPAT1 possesses enzymatic activity, the three-dimensional structure of OsPAT1.1, the main product of *OsPAT1*, were predicted by AlphaFold 3 to compare with that of PAT1. Interestingly, OsPAT1.1 and PAT1 possessed nearly identical structures, except for their N-termini (Figure S8a–c). Moreover, their structures perfectly resembled the reported crystal structure of yeast TRP4 (Figure 4a and Figure S8a,d). The core structures of both OsPAT1.1 and PAT1 consisted of an α-helical domain and an α/β domain (Figure S8b,c). A cleft was formed between the two domains in OsPAT1.1, similar to that in TRP4 for binding the substrate PRPP (Figure S8b,d) [38]. To reveal the possible binding conformation, AutoDock was used to predict the potential interactions between OsPAT1.1 and PRPP (Figure 4b). In this conformation, ten residues in total could interact with PRPP, and six of them were located in the reported PRPP-binding motifs in TRP4 (Figure 4b,c). This finding suggested that OsPAT1 may function as an AnPRT enzyme.

To test the enzyme activities, OsPAT1.1 and PAT1 were ectopically expressed in the yeast *trp4* mutant strain. Surprisingly, full-length OsPAT1.1 failed to complement the *trp4* mutant on tryptophan-deficient medium, while PAT1 succeeded (Figure 4d,e). It was reported that PAT1 contained a plastid signal sequence at its N-terminus [33]. However, only a short undefined signal sequence predicted by InterPro were found in OsPAT1.1 or OsPAT1.2 (Figure 4d). Therefore, the plastid signal sequence of PAT1 was fused to OsPAT1.1 in further experiments. And the modified OsPAT1.1 successfully rescued the *trp4* mutant (Figure 4d,e), indicating that OsPAT1 possessed functional AnPRT activity in yeast, though its function might depend on localization signals.

### 2.5. Expression Pattern and Protein Subcellular Localization of OsPAT1

**Figure 5:**
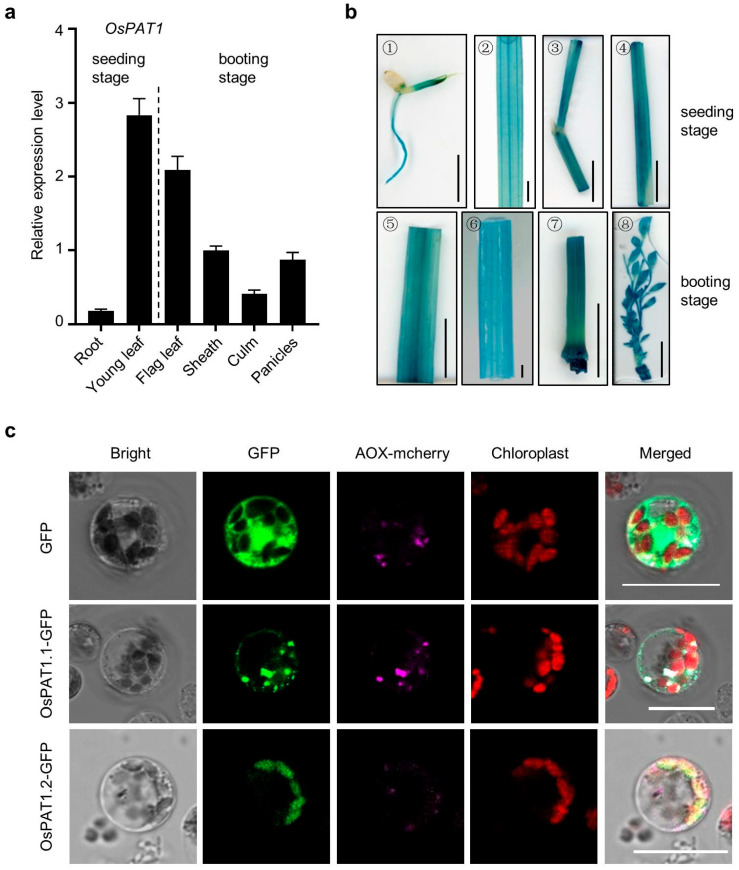
Express pattern and protein subcellular localizations of *OsPAT1*. (**a**) Relative expression levels of *OsPAT1* in different organs of WT plants. Data were presented as means ± SD of three biological replicates. (**b**) Histochemical analysis of pLMM9::GUS transgenic plants. GUS signals were detected in germinated seeds (1), leaves, pulvinus, and sheathes of young seedlings (2–4), flag leaves, sheathes, culms, and panicles at booting stage (5–8). Bars = 1 cm. (**c**) Subcellular localizations of OsPAT1.1 and OsPAT1.2. GFP was used as the control. AOX-mcherry was used as the maker for mitochondria localization. Bars = 20 µm.

The expression of *OsPAT1* was analyzed in different tissues at seedling and adult stages. High expression levels of *OsPAT1* were found in young leaves and flag leaves. *OsPAT1* was also expressed in sheaths, panicles, and culms, while its expression in roots was very low (Figure 5a). Then, the promoter of *OsPAT1* was linked with the β-glucuronidase (GUS) reporter gene and transformed into MDJ8. The GUS reporter was detected in many tissues, including radicles, leaves, pulvinus, and sheaths at the seeding stage, and flag leaves, sheathes, culms, and panicles at the booting stage (Figure 5b). These results indicated that *OsPAT1* could express in most rice tissues.

To determine the subcellular localization of OsPAT1, GFP was fused to both OsPAT1.1 and OsPAT1.2 variants. Surprisingly, OsPAT1.2-GFP was localized in chloroplasts, whereas OsPAT1.1-GFP was localized in mitochondria (Figure 5c). To further validate these findings, fragments of OsPAT1.1 and OsPAT1.2 were analyzed (Figure S9a). The N-terminus of OsPAT1 was localized in chloroplasts, while the remaining regions of both OsPAT1.1 and OsPAT1.2 were localized in mitochondria (Figure S9b). These results suggested that the N-terminus of OsPAT1 alone was insufficient to function as a chloroplast-targeting signal peptide and requires the last six amino acids of OsPAT1.2 for proper localization.

### 2.6. The Expression of Many Tryptophan Synthesis Pathway Genes Was Induced in the lmm9 Mutant

**Figure 6:**
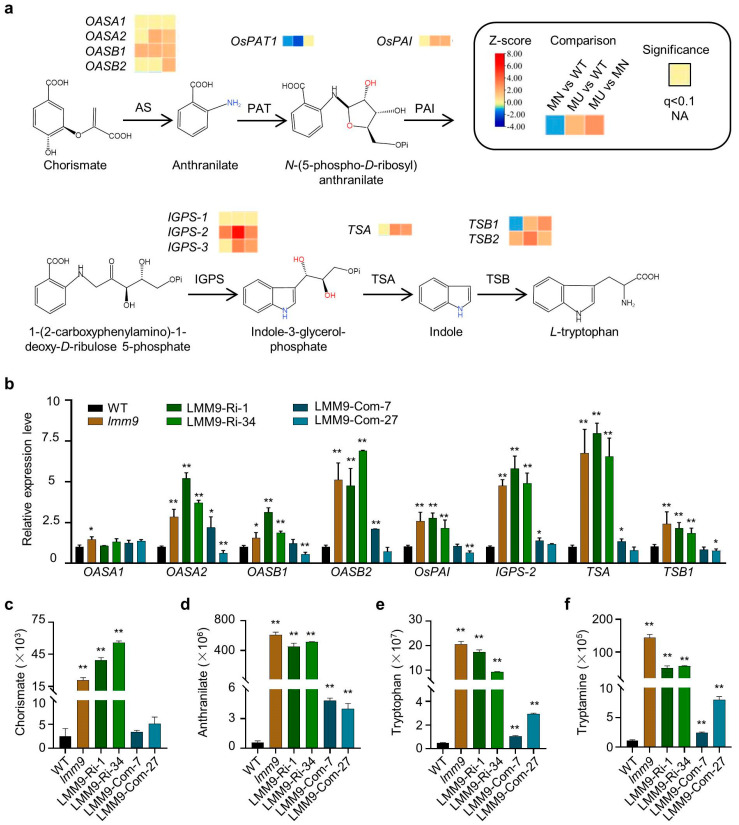
The expressions of several genes in tryptophan synthesis pathway were affected by *OsPAT1* expression. (**a**) Heat maps of the expressions of tryptophan synthesis pathway genes in RNA-seq analysis. AS, anthranilate synthase; PAT, phosphoribosylanthranilate transferase; PAI, N-(5′-phosphoribosyl)anthranilate isomerase; IGPS, indole-3-glycerolphosphate synthase; TSA, tryptophan synthase α subunit; TSB, tryptophan synthase β subunit. (**b**) Relative expression levels of tryptophan synthesis pathway genes in WT, *lmm9*, and transgenic plants. Data were shown as means ± SD of three replicates. (**c**–**f**) Relative contents of chorismate (**c**), anthranilate (**d**), tryptophan (**e**), and tryptamine (**f**) in WT, *lmm9* and related transgenic plants. The significance compared with WT was determined by Student’s *t*-test at *p* < 0.05 (*) and *p* < 0.01 (**).

To identify which genes were influenced in the *lmm9* mutant, total RNA from rice leaves was sequenced. Compared with the WT, the expression of genes participating in metabolic pathways and the biosynthesis of secondary metabolites were significantly altered in the *lmm9* mutant (Figure S10). Notably, the expression of many tryptophan synthesis pathway genes changed similarly in *lmm9* (Figure 6a). Several genes involved in downstream reactions of *OsPAT1*, including *OsPAI*, *IGPS-2*, *TSA*, and *TSB1*, were all induced in *lmm9* (Figure 6a). This could be easily understood since PRA production was possibly blocked in *lmm9*, and induction of these genes would compensate for it. However, the genes encoding the α and β subunits of AS, except *OASA1*, were also increased in *lmm9* (Figure 6a). These results collectively suggested that a significant metabolic disorder probably occurred in *lmm9*, similar to the phenomenon observed in the rice *els1* and *ell1* mutant [26,27]. The expression results were further validated using LMM9-Com and LMM9-Ri plants. Significant upregulation of *OASA2*, *OASB1*, *OASB2*, *OsPAI*, *IGPS-2*, *TSA*, and *TSB1* was observed in LMM9-Ri plants, similar to the *lmm9* mutants. In contrast, the expression of the same genes in LMM9-Com plants remained unaffected (Figure 6b).

Moreover, the expression of genes that encoded enzymes at downstream of the tryptophan synthesis pathway was also affected. For example, the gene expression of *TDC1*, *TDC3*, *T5H* (*ELL1/SL*), and *OsCOMT15*, which are involved in the serotonin and melatonin synthesis, was all induced in *lmm9* and LMM9-Ri plants, compared with the WT and LMM9-Com plants (Figure S11).

To further confirm whether the induction of these genes would affect the synthesis and degradation of tryptophan, the relative contents of tryptophan and several important metabolites in *lmm9* and related transgenic plants were measured. Chorismate, which participates upstream of OsPAT1, was accumulated in *lmm9* and LMM9-Ri plants compared to those in WT and LMM9-Com plants (Figure 6c). Anthranilate, the substrate of OsPAT1, was also accumulated in *lmm9* and LMM9-Ri plants, compared to WT (Figure 6d). Similarly, both tryptophan and tryptamine levels were significantly increased in *lmm9* and LMM9-Ri plants (Figure 6e,f). Meanwhile, the contents of anthranilate, tryptophan, and tryptamine were slightly upregulated in LMM9-Com plants, possibly due to the higher *OsPAT1* expression mentioned above (Figure 6d,f). All these results indicated that both tryptophan anabolism and catabolism were increased in *lmm9*.

## 3. Discussion

LMMs are valuable materials for studying plant cell death and defense responses. The genes responsible for LMMs vary widely. Here, we report the characterization and identification of a new rice LMM, *lmm9*, which exhibited significant lesion mimic phenotypes in multiple tissues and a high lethality rate. It was found that downregulation of *OsPAT1*, the sole homolog of *Arabidopsis PAT1* in the rice genome, caused the *lmm9* phenotype. And similar lesion mimics were observed in *OsPAT1* knockdown and knockout rice plants. However, the *Arabidopsis trp1-1* mutant, which carries the defective *PAT1* gene, is a well-known tryptophan auxotroph. Although the development and growth of *trp1-1* plants were impaired, no lesion mimic phenotype was observed. This phenomenon reflects the functional differences between the two homologs in rice and *Arabidopsis*.

The N-terminal sequence of OsPAT1 was different from that of PAT1, and the subcellular location of its main product, OsPAT1.1, also differed from that of PAT1. By searching the database, it was found that the N-termini of AnPRT homologs in monocots were highly conserved, and no homologs contained the plastid transit peptide of PAT1 (Figure S9). These results suggested that AnPRT homologs in monocots and dicots might have diverged during evolution, raising a possibility that PRA could be generated in different organelles depending on the species. Actually, tryptophan can also be synthesized in microbes, which lack chloroplasts. Therefore, more studies are needed to fully elucidate whether tryptophan can be synthesized in other organelles in different plant species.

For a long time, the link between tryptophan and LMM received little attention. Although the *sl* mutant was the first LMM in rice, the *sl* gene was not among the first cloned rice LMM genes. The *sl* and its allelic mutant, *ell1*, exhibited lesions similar to those in *lmm9*, and the formation could be influenced by light [23,25]. Both mutants were caused by mutations in the tryptamine 5-hydroxylase, which catalyzes tryptamine conversion to serotonin [24,25]. It was found that both tryptophan synthesis and catabolism genes were induced and the tryptophan metabolism was disturbed in *ell1* and *sl* mutants [23,26]. Unlike the *ELL1/SL* gene functions in tryptophan catabolism, the *ELS1/ASA2* gene encodes the α subunit of the AS enzyme and functions in tryptophan synthesis. In *els1*, the mutation in *ELS1/ASA2* triggered the induction of its homolog, *ASA1*, and other tryptophan synthesis pathway genes as well [27]. The levels of tryptophan and many related metabolites were also increased, leading to metabolic dysregulation in *els1*. Suppressing *ASA1* expression restored the lesion mimic phenotype in *els1* [27]. In all the three mutants, a common character is the constitutive activation of tryptophan metabolism. In *lmm9*, the expression of genes involved in tryptophan synthesis and degradation was also induced, resulting in significant accumulation of tryptophan and other related metabolites. As a consequence, the lesion mimic phenotype in *lmm9* probably resulted from tryptophan metabolic disorder.

Tryptophan synthesis is a multi-step process. The enzymes involved in this process are encoded by many different genes. However, the regulation of their expression has been less studied. In *els1*, the increased expression of *ASA1* could be caused by the compensation response to the mutation of *ELS1/ASA2*, while the mechanism of the induction of other tryptophan synthesis pathway genes remains unclear [27]. In both *lmm9* and *ell1/sl*, the expression of several tryptophan synthesis pathway genes was also simultaneously induced. This phenomenon suggested that there might be a coordinated regulatory mechanism in rice to maintain synchronized expression of these genes at the transcriptional level, which differed from feedback regulation at the protein level [39]. This mechanism might enable plants to respond effectively to environmental changes or stresses [23,25]. However, such kind of transcriptional activation of tryptophan synthesis pathway genes had not been reported in related *Arabidopsis* mutants. As a result, further studies are needed to fully clarify the regulatory mechanism and the differences between rice and *Arabidopsis*. In addition, the tryptophan contents are significantly increased and the tryptophan biosynthesis pathway genes are significantly upregulated in *lmm9* compared with WT, however it lacks direct evidences linking tryptophan to the lesion mimic formation. Therefore, the potential relationship between tryptophan and the lesion mimic formation needs to be elucidated through further analysis, such as comparing the lesion mimic phenotype after applying exogenous tryptophan and/or tryptophan inhibitor in the WT and mutants.

## 4. Materials and Methods

### 4.1. Plant Materials and Growth Conditions

The wildtype rice (*O. sativa* ssp. *japonica/geng*) used in this study was *japonica* cultivar Mudanjiang 8 (MDJ8). All plants were cultivated under standard field conditions during the typical growing season at Wuhan experimental station, China (geographical coordinates: 114.2° E Longitude, 30.5° N latitude; elevation 15 M above sea level; average daily temperature range 28–30 °C).

### 4.2. Trypan Blue Staining

Equivalent leaf segments of WT and *lmm9* mutant were selected at 30 days post-germination. The samples were subjected to vacuum infiltration in a trypan blue staining solution to ensure complete tissue penetration. Following a 2 min boiling, samples were incubated at 37 °C for overnight. Post-incubation, stained tissues were decolorated in chloral hydrate solution until achieving optimal optical clarity. The characteristic of stained tissues was observed with stereo microscopy.

### 4.3. Physiological Measurements

Malondialdehyde (MDA) content in WT and *lmm9* mutant leaves was quantified using the thiobarbituric acid (TBA) colorimetric method [40]. Absorbance values of the reaction mixtures were measured at 532 nm, 600 nm, and 450 nm using spectrophotometer, with subsequent calculation of MDA concentration based on MDA content formula.

Superoxide dismutase (SOD) activity of WT and *lmm9* mutant leaves was determined according to the manufacturer’s protocol for the Superoxide Dismutase Activity Assay Kit (Solarbio). Absorbance at 560 nm were measured via spectrophotometer to calculate enzymatic activity using the SOD activity formula.

Peroxidase (POD) activity was measured through guaiacol-based colorimetric analysis. SOD activity was calculated by measuring absorbance at 470 nm at 30 s intervals absorbance values recording.

Catalase (CAT) activity was determined by monitoring the absorbance decline at 240 nm at 30 s intervals absorbance values recording [41], calculation of CAT activity based on formula.

### 4.4. Mapping of the lmm9 Gene

Due to the failure of crossing *lmm9* mutant with the *indica* cultivar, Minghui 63 (MH63), the normal plants segregate alongside *lmm9* mutants from the same line were chosen as the female parents. According to Mendelian inheritance, these phenotypically normal plants had a two-thirds probability of carrying the *lmm9* gene. These plants were hybridized with MH63, and both hybrids and self-pollinated seeds were harvested. Subsequently, the self-pollinated progeny were cultivated and checked for lesion mimic phenotypes to verify whether their parent plants harbored *lmm9* gene. Lesions were found in one family, indicating its parent plant was heterozygous. Then, the hybrids from the same plant was cultivated and self-crossed separately to generate segregating populations, since only half of them carried the *lmm9* gene. Two populations were obtained, and both were planted in the normal growing season, with one carrying the *lmm9* gene for mapping. In this population of about two thousand plants, those that developed lesions were used to identify the *lmm9* gene. 105 SSR markers were used in the preliminary mapping, and the *lmm9* gene was localized to a 3 Mb region between RM3202 and RM3117 on chromosome 3. After adding new SSR markers and plant samples, *lmm9* was fine-mapped to a 269 kb region between RM3372 and RM14341.

Then, genomic DNA was extracted from WT and *lmm9* mutant plants at the seedling stage, and whole-genome sequencing library of them were prepared following established protocols [42] and sequenced using Illumina X10 platform (San Diego, CA, USA). The sequencing reads were mapped to the region between RM3372 and RM14341 to identify the differences between WT and *lmm9* mutant.

### 4.5. Vector Construction and Transformation

In the complementation test, an 8.6 kb fragment containing the entire 4.1 kb genomic sequence of *Os03g03450*, along with 2.1 kb upstream and 2.5 kb downstream region, was amplified using MDJ8 genomic DNA as the template. This fragment was ligated into the pCAMBIA2300 vector via homologous recombination.

For RNAi vector construction, a 310 bp fragment amplified from MDJ8 cDNA was inserted into the pDS1301 vector.

For knockout vector construction, CRISPR-P 2.0 (http://crispr.hzau.edu.cn/CRISPR2, accessed on 5 July 2021) was used to design two targets located in the second exon (5′-*gctgctggaggaggagaacgagg*-3′) and the third exon (5′-*gggttggcgaaggcgatgatagg*-3′) [43]. The adapter primers were designed based on these targets, and the target gRNA expression cassettes were inserted into the Cas9 vector using the Liu Yaoguang system to construct the gene editing vector [44].

For GUS vector construction, a 2.3 kb promoter region of *Os03g03450* was amplified using MDJ8 genomic DNA as the template. This fragment was ligated into the DX2181 vector.

The vectors were sent to Wuhan Tianwen Company (Wuhan, China) for rice transformation. The *lmm9* mutant was used as the recipient of the complementation vector, and MDJ8 was used for the other vectors. The transgenic plants were analyzed using specific primers (Table S1).

### 4.6. Protein Sequence and Phylogenetic Analysis

The OsPAT1 protein sequence was employed as the query in the NCBI blastp program to identify homologous proteins (Table S2). The retrieved protein sequences were compared using ClustalX version 2, and a phylogenetic tree was constructed with MEGA 11 software through the Neighbor-Joining method. To analyze conserved amino acid residues, homologs from *Oryza sativa*, *Triticum aestivum*, *Zea mays*, *Arabidopsis thaliana*, *Brassica napus*, *Physcomitrium patens*, *Fragilariopsis cylindrus*, and *Chloroflexi bacterium* were compared using GENEDOC 2.7 software.

### 4.7. Subcellular Location of OsPAT1

The alternatively spliced transcripts of OsPAT1 were amplified using specific primers (Table S1). The resulting fragments were ligated into the pAN580-GFP vector via homologous recombination. These constructs, along with a mitochondrial marker vector and a control vector (35S-GFP), were co-transfected into rice protoplasts. Fluorescence signals were captured using a Zeiss LSM980 (Zeiss, Baden-Württemberg, Germany) laser scanning confocal microscope.

### 4.8. Protein Structure Prediction

The three-dimensional structures of PAT1 and OsPAT1 were modeled using AlphaFold 3 (https://www.alphafoldserver.com, accessed on 20 June 2024) [45].

### 4.9. Molecular Docking

The interactions between OsPAT1 and PRPP was predicted using PyMOL 3 and AutoDockTools-1.5.7 software [46].

### 4.10. RNA-Seq Analysis

Leaf samples were collected from three groups: WT, *lmm9* mutants with lesion mimics (MU), and *lmm9* mutants without lesion mimics (MN), each with three biological replicates. Total RNA was extracted and submitted to the Shenzhen Institute of Agricultural Genomics, Chinese Academy of Agricultural Sciences for high-throughput sequencing. The resulting sequencing data were analyzed to identify differentially expressed genes (DEGs) defined by thresholds of |Log_2_(fold change)| ≥ 1 and false discovery rate (FDR) ≤ 0.05), each with in MN vs. WT, MU vs. WT, and MN vs. MU groups were screened, and their distribution was visualized in a Venn diagram based on transcriptomic data. Kyoto Encyclopedia of Genes and Genomes (KEGG) pathway enrichment analysis (FDR < 0.05) was subsequently performed on the identified DEGs.

### 4.11. qRT-PCR Analysis

The primers used for qRT-PCR are listed in Table S1. For each target gene, reactions were performed with three technical replicates, and independent experiments were repeated at least twice. Representative data from one experimental repetition are shown. Relative gene expression levels normalized to the control are presented as column graphs.

### 4.12. Metabolites Analysis

The WT, *lmm9*, LMM9-Com, and LMM9-RNAi plants were all grown under standard condition. Their flag leaves were collected at booting stage for metabolites analysis. Three biological replicates were collected for each sample. The samples were extracted and analyzed following the methods previously reported [47].

### 4.13. Accession Numbers

The sequence data of *OsPAT1* can be found in the GenBank through accession numbers PQ660886.1.

## Data Availability

The original contributions presented in this study are included in the article/Supplementary Material and openly available in Genebank through accession numbers PQ660886.1. Further inquiries can be directed to the corresponding author(s).

## Supplementary Materials

The following supporting information can be downloaded at: https://www.mdpi.com/article/10.3390/ijms26199428/s1.

## Abbreviations

The following abbreviations are used in this manuscript:

LMMs	Lesion mimic mutants
HR	Hypersensitive response
PCD	Programmed cell death
ROS	reactive oxygen species
UROD	Uroporphyrinogen decarboxylase
PEPP	Phosphoribosyl group from phosphoribosyl-α1-pyrophosphate
Ppi	Pyrophosphate
PRA	N-(5′-phosphoribosyl)anthranilate
AnPRT	Anthranilate phosphoribosyltransferase
PRT	Phosphoribosylanthranilate phosphoribosyltransferase
*PAT1*	*Phosphoribosylanthranilate transferase 1*
WT	Wide type
CAT	Catalase
POD	Peroxidase
SOD	Superoxide dismutase
