# Supplementary material for: Decreased Expression of a Phosphoribosylanthranilate Transferase-Encoding Gene, OsPAT1, Causes Lesion Mimics in Rice
Source: Int J Mol Sci. 2025 Sep 26;26(19):9428. doi: 10.3390/ijms26199428 (PMC12524759; doi:10.3390/ijms26199428)
Supplement: Supplementary file 1 [file ijms-26-09428-s001.zip › ijms-3886559-supplementary.pptx]

## Slide 1
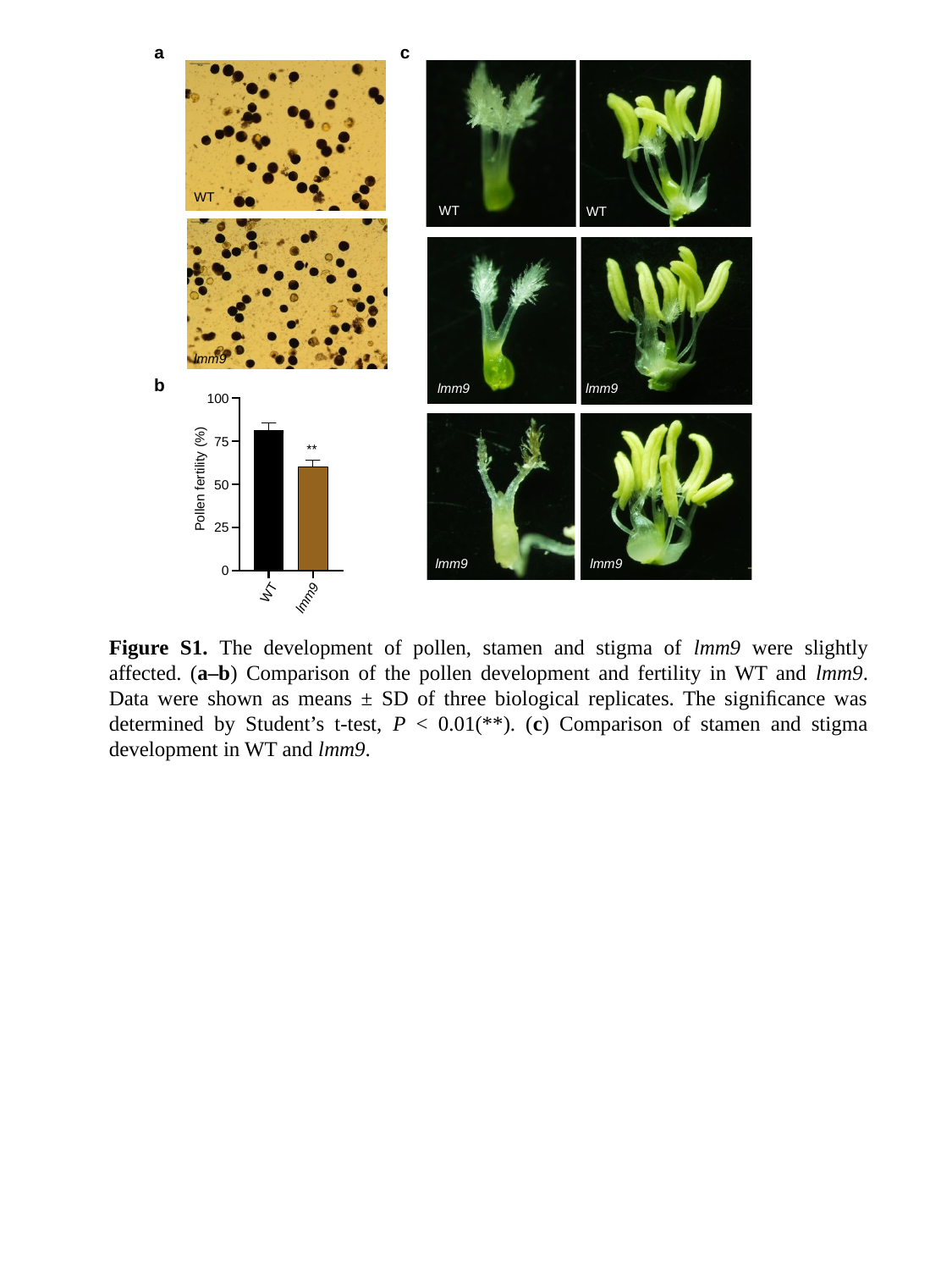

a
c
WT
WT
WT
lmm9
b
lmm9
lmm9
100
75
**
Pollen fertility (%)
50
25
0
WT
lmm9
lmm9
lmm9
lmm9
Figure S1. The development of pollen, stamen and stigma of lmm9 were slightly affected. (a–b) Comparison of the pollen development and fertility in WT and lmm9. Data were shown as means ± SD of three biological replicates. The signiﬁcance was determined by Student’s t-test, P < 0.01(**). (c) Comparison of stamen and stigma development in WT and lmm9.

## Slide 2
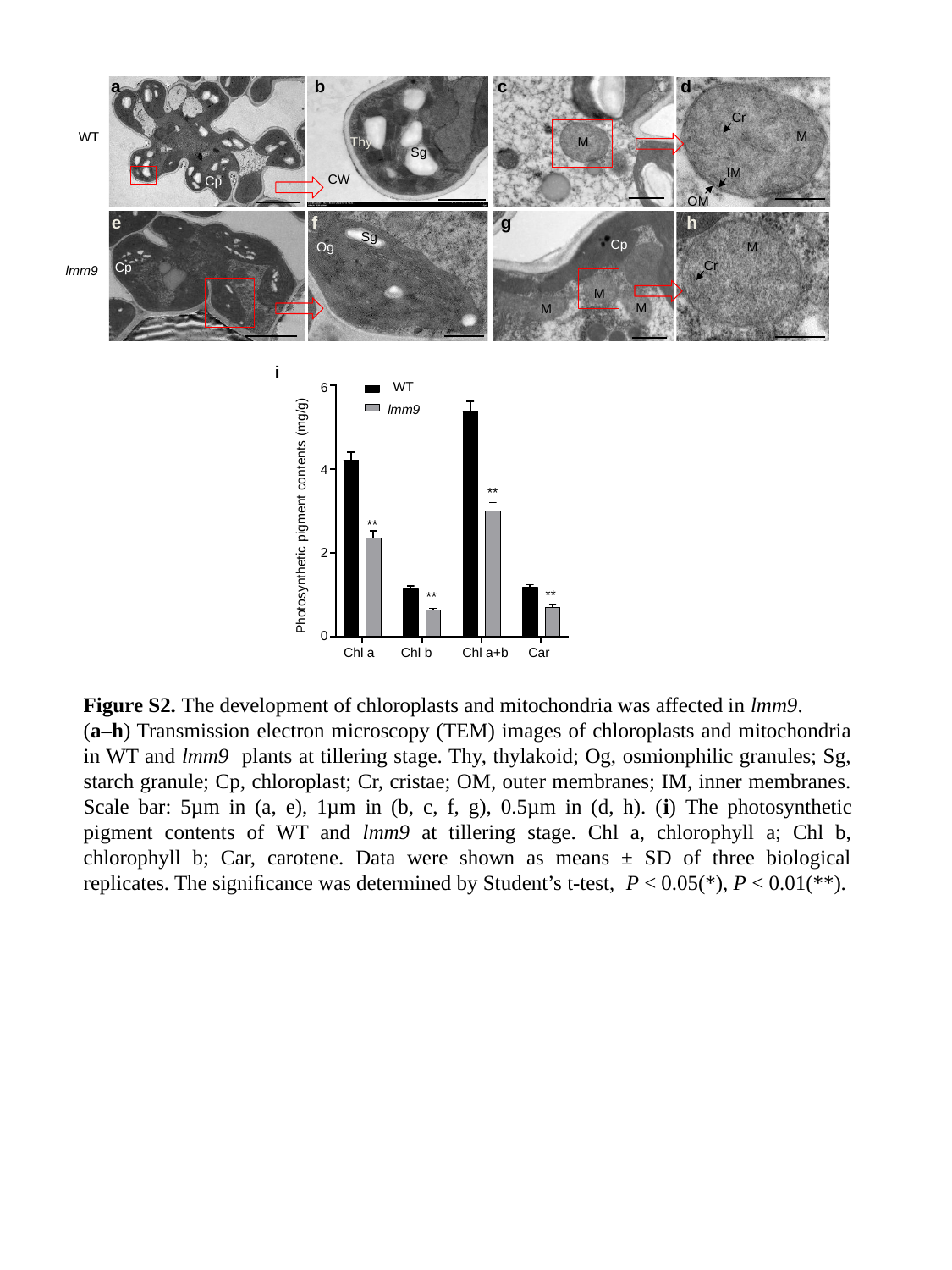

a
b
c
d
Cr
M
WT
M
Thy
Sg
IM
CW
Cp
OM
e
f
g
h
Sg
Cp
Og
M
Cr
Cp
lmm9
M
M
M
i
WT
6
lmm9
4
**
Photosynthetic pigment contents (mg/g)
**
2
**
**
0
Chl a
Chl b
Chl a+b
Car
Figure S2. The development of chloroplasts and mitochondria was affected in lmm9.
(a–h) Transmission electron microscopy (TEM) images of chloroplasts and mitochondria in WT and lmm9 plants at tillering stage. Thy, thylakoid; Og, osmionphilic granules; Sg, starch granule; Cp, chloroplast; Cr, cristae; OM, outer membranes; IM, inner membranes. Scale bar: 5µm in (a, e), 1µm in (b, c, f, g), 0.5µm in (d, h). (i) The photosynthetic pigment contents of WT and lmm9 at tillering stage. Chl a, chlorophyll a; Chl b, chlorophyll b; Car, carotene. Data were shown as means ± SD of three biological replicates. The signiﬁcance was determined by Student’s t-test, P < 0.05(*), P < 0.01(**).

## Slide 3
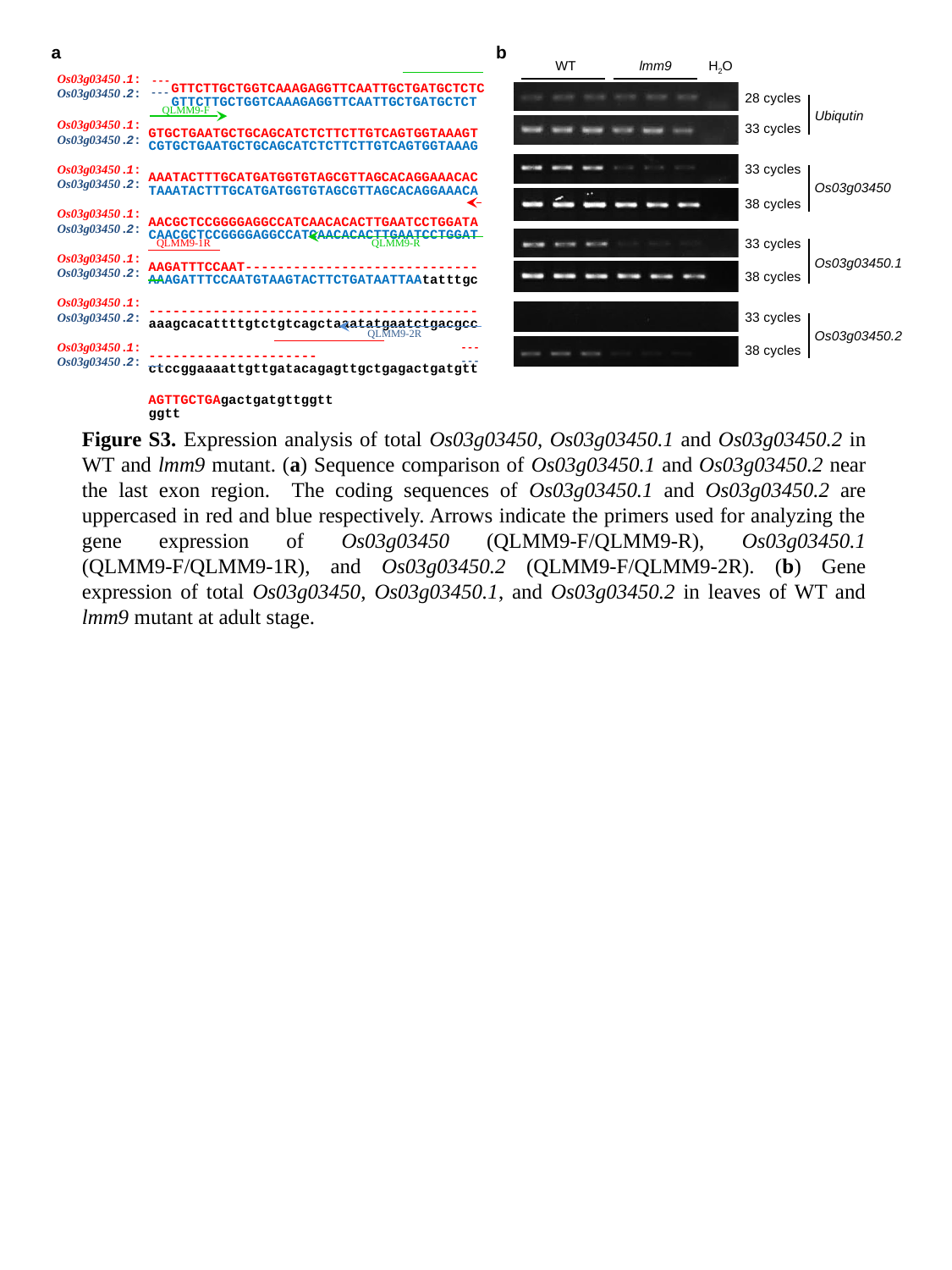

a
b
GTTCTTGCTGGTCAAAGAGGTTCAATTGCTGATGCTCTCGTGCTGAATGCTGCAGCATCTCTTCTTGTCAGTGGTAAAGTAAATACTTTGCATGATGGTGTAGCGTTAGCACAGGAAACACAACGCTCCGGGGAGGCCATCAACACACTTGAATCCTGGATAAAGATTTCCAAT-------------------------------------------------------------------------------------------AGTTGCTGAgactgatgttggtt
WT
lmm9
H2O
GTTCTTGCTGGTCAAAGAGGTTCAATTGCTGATGCTCTCGTGCTGAATGCTGCAGCATCTCTTCTTGTCAGTGGTAAAGTAAATACTTTGCATGATGGTGTAGCGTTAGCACAGGAAACACAACGCTCCGGGGAGGCCATCAACACACTTGAATCCTGGATAAAGATTTCCAATGTAAGTACTTCTGATAATTAAtatttgcaaagcacattttgtctgtcagctaaatatgaatctgacgccctccggaaaattgttgatacagagttgctgagactgatgttggtt
 ---
Os03g03450 .1:
Os03g03450 .2:
 ---
28 cycles
QLMM9-F
Ubiqutin
Os03g03450 .1:
Os03g03450 .2:
33 cycles
33 cycles
Os03g03450 .1:
Os03g03450 .2:
Os03g03450
38 cycles
Os03g03450 .1:
Os03g03450 .2:
33 cycles
QLMM9-R
QLMM9-1R
Os03g03450 .1:
Os03g03450 .2:
Os03g03450.1
38 cycles
Os03g03450 .1:
Os03g03450 .2:
33 cycles
QLMM9-2R
Os03g03450.2
 ---
Os03g03450 .1:
Os03g03450 .2:
38 cycles
 ---
Figure S3. Expression analysis of total Os03g03450, Os03g03450.1 and Os03g03450.2 in WT and lmm9 mutant. (a) Sequence comparison of Os03g03450.1 and Os03g03450.2 near the last exon region. The coding sequences of Os03g03450.1 and Os03g03450.2 are uppercased in red and blue respectively. Arrows indicate the primers used for analyzing the gene expression of Os03g03450 (QLMM9-F/QLMM9-R), Os03g03450.1 (QLMM9-F/QLMM9-1R), and Os03g03450.2 (QLMM9-F/QLMM9-2R). (b) Gene expression of total Os03g03450, Os03g03450.1, and Os03g03450.2 in leaves of WT and lmm9 mutant at adult stage.

## Slide 4
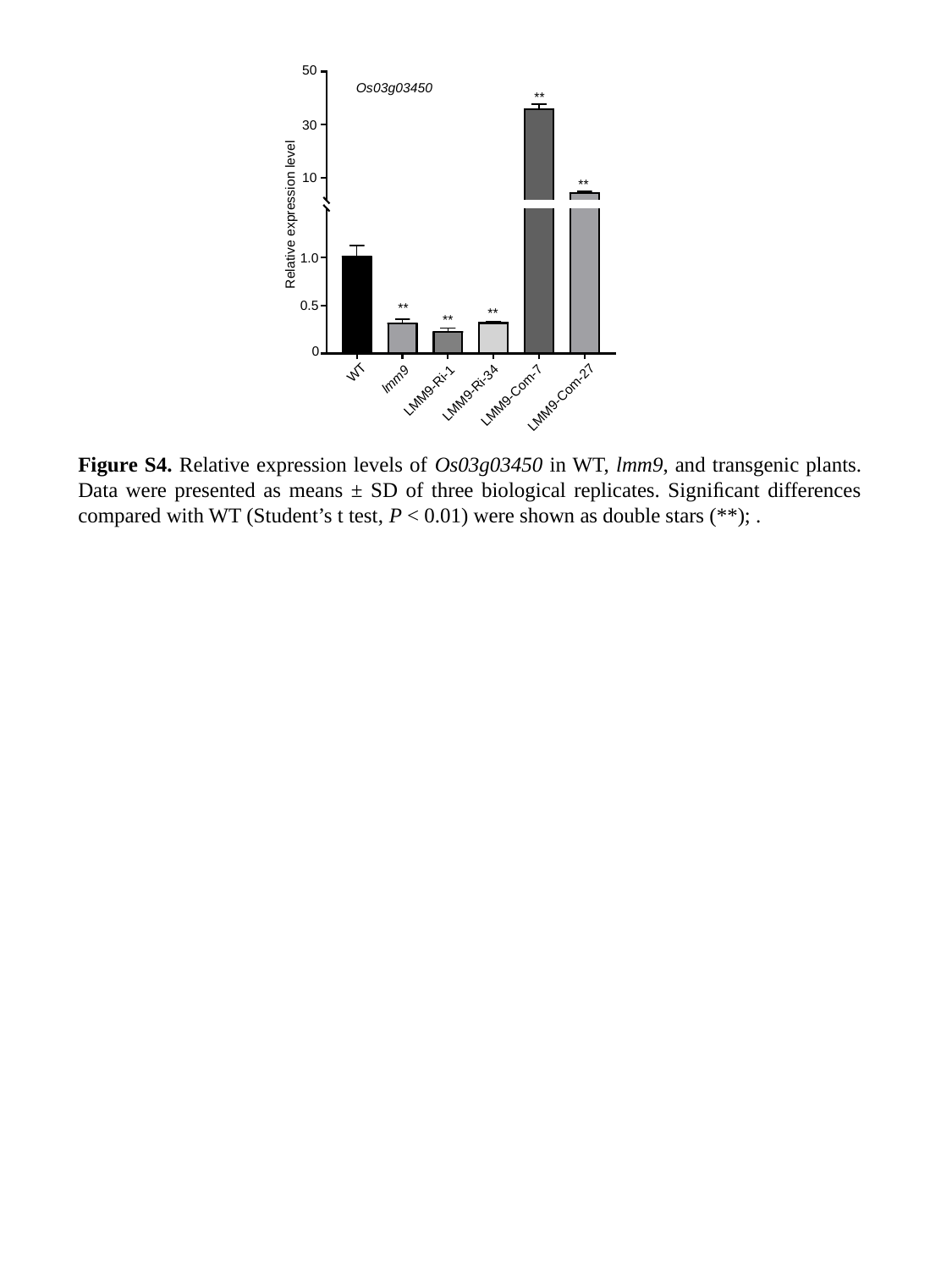

50
Os03g03450
**
30
10
**
Relative expression level
1.0
0.5
**
**
**
0
WT
lmm9
LMM9-Ri-1
LMM9-Ri-34
LMM9-Com-7
LMM9-Com-27
Figure S4. Relative expression levels of Os03g03450 in WT, lmm9, and transgenic plants. Data were presented as means ± SD of three biological replicates. Signiﬁcant differences compared with WT (Student’s t test, P < 0.01) were shown as double stars (**); .

## Slide 5
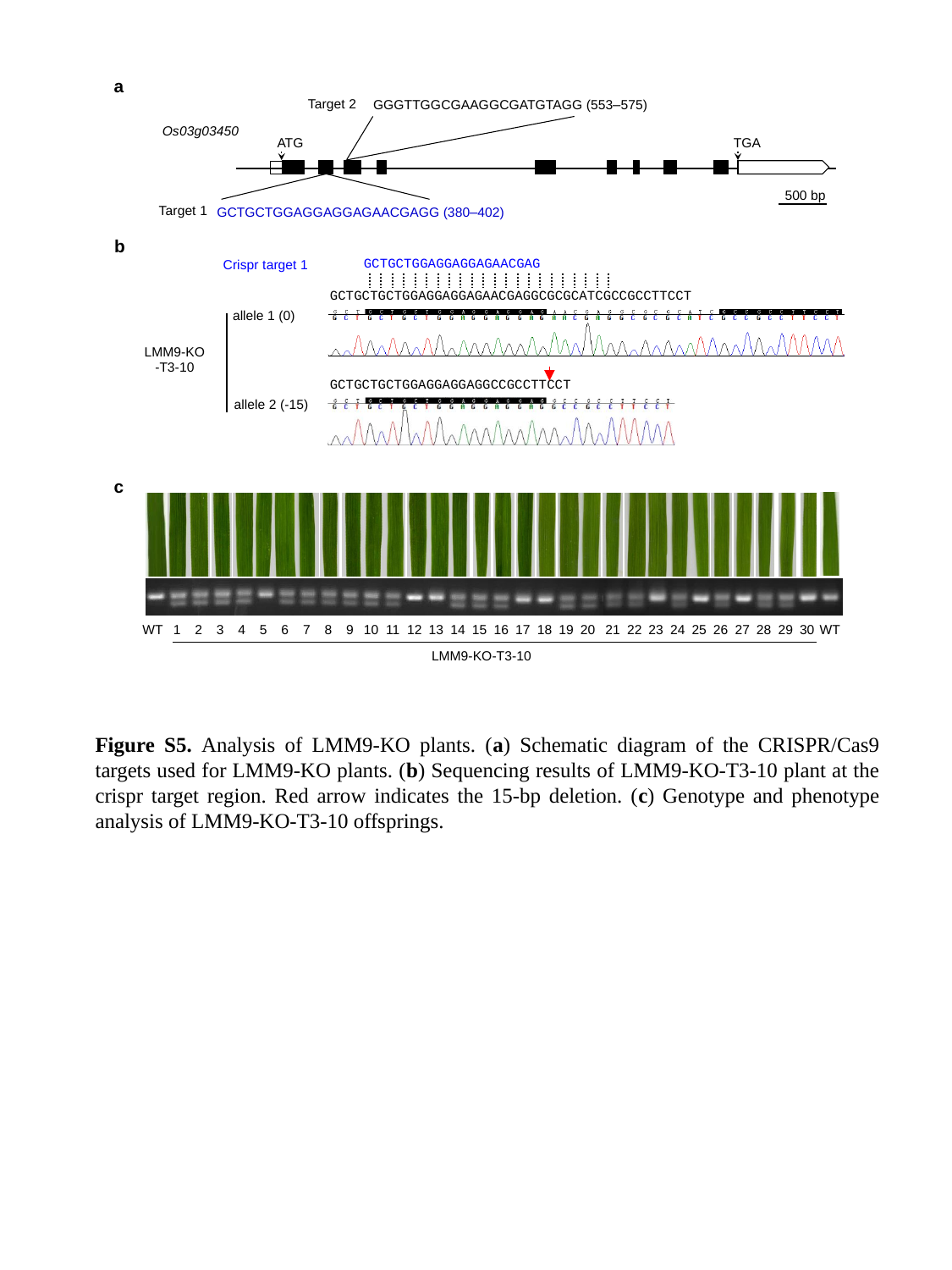

a
Target 2
GGGTTGGCGAAGGCGATGTAGG (553–575)
Os03g03450
ATG
TGA
500 bp
Target 1
GCTGCTGGAGGAGGAGAACGAGG (380–402)
b
GCTGCTGGAGGAGGAGAACGAG
Crispr target 1
GCTGCTGCTGGAGGAGGAGAACGAGGCGCGCATCGCCGCCTTCCT
allele 1 (0)
LMM9-KO
-T3-10
GCTGCTGCTGGAGGAGGAGGCCGCCTTCCT
allele 2 (-15)
c
WT
1
2
3
4
5
6
7
8
9
10
11
12
13
14
15
16
17
18
19
20
21
22
23
24
25
26
27
28
29
30
WT
LMM9-KO-T3-10
Figure S5. Analysis of LMM9-KO plants. (a) Schematic diagram of the CRISPR/Cas9 targets used for LMM9-KO plants. (b) Sequencing results of LMM9-KO-T3-10 plant at the crispr target region. Red arrow indicates the 15-bp deletion. (c) Genotype and phenotype analysis of LMM9-KO-T3-10 offsprings.

## Slide 6
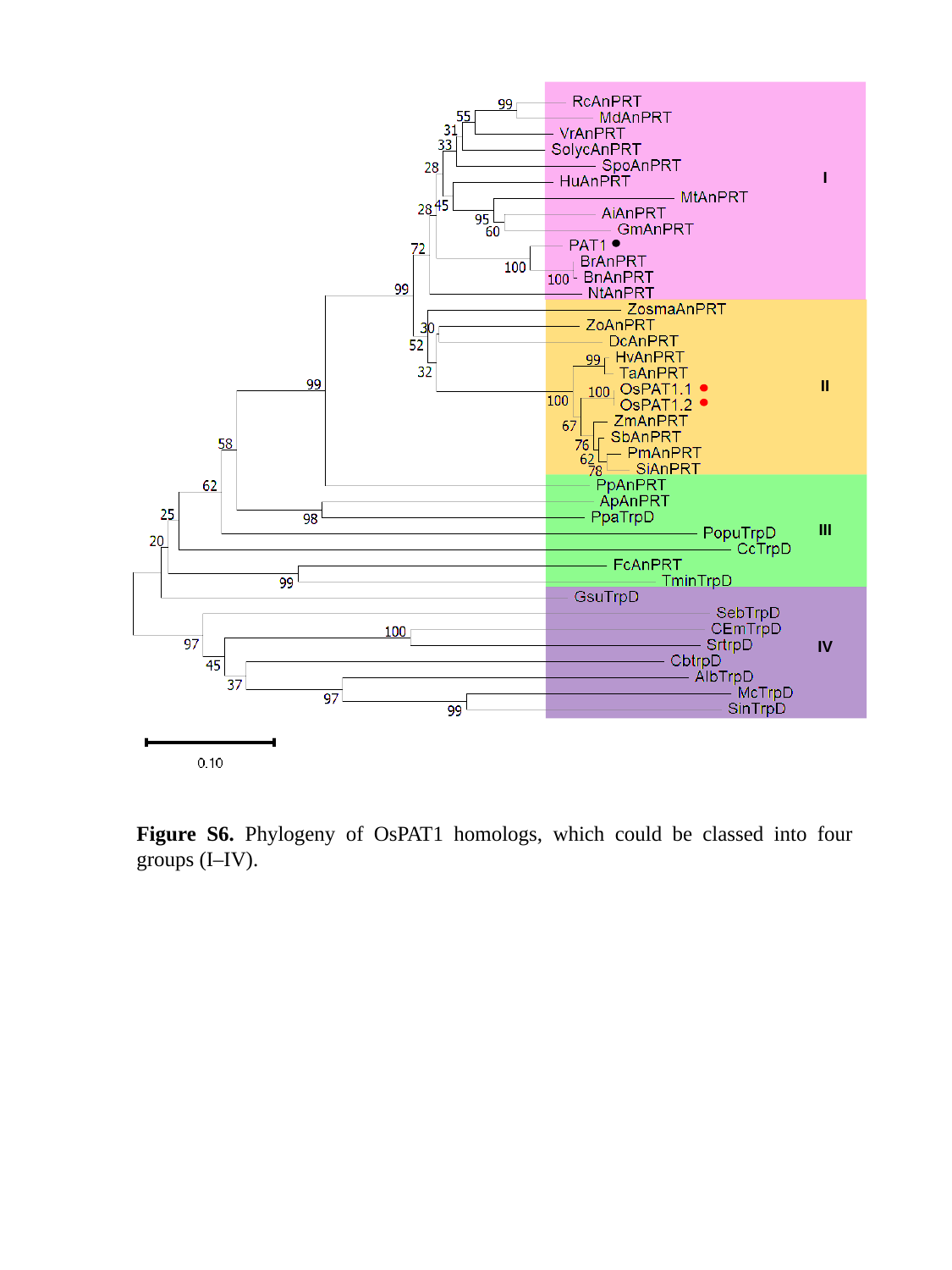

I
II
III
IV
Figure S6. Phylogeny of OsPAT1 homologs, which could be classed into four groups (I–IV).

## Slide 7
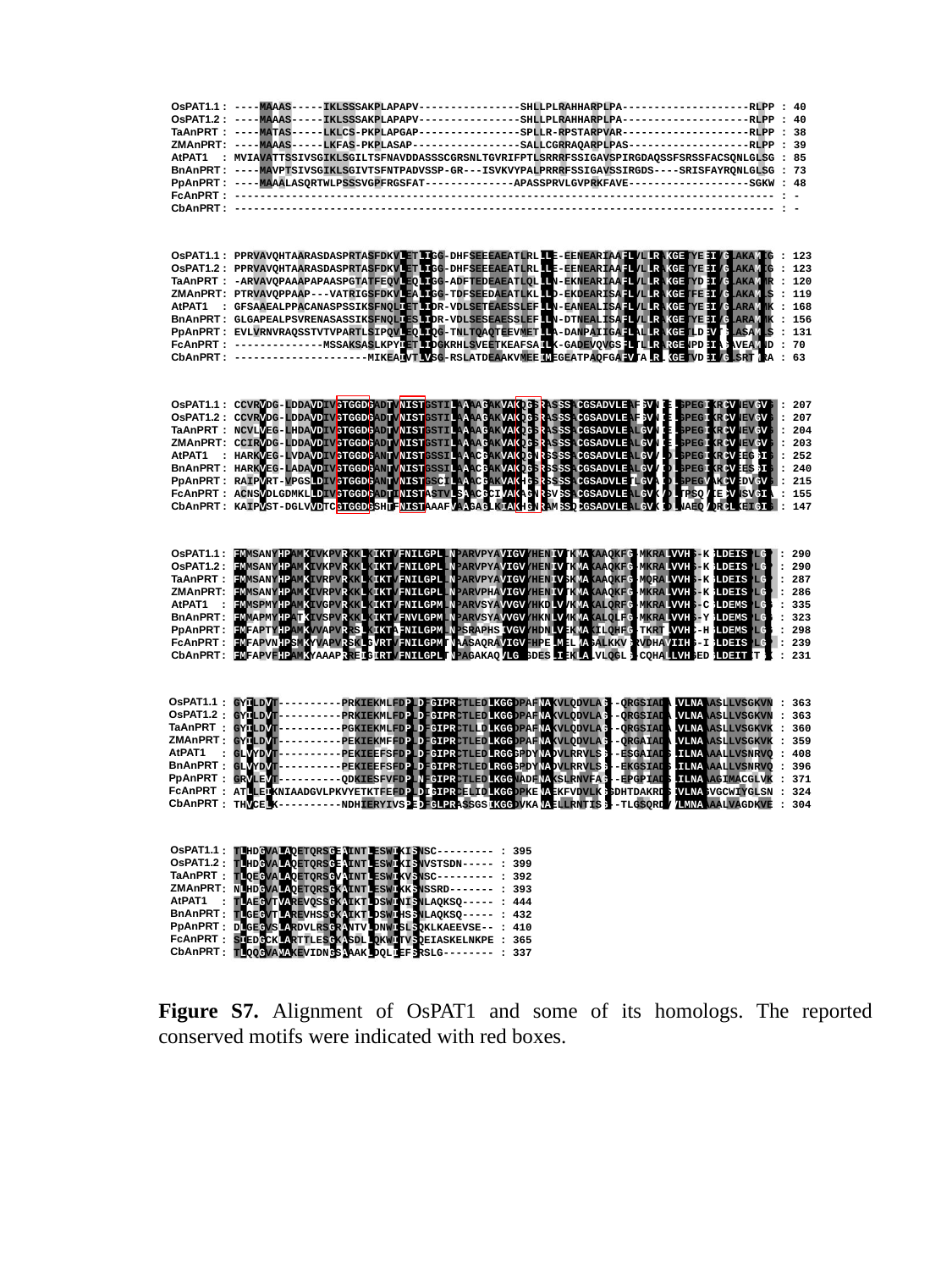

OsPAT1.1
OsPAT1.2
TaAnPRT
ZMAnPRT
AtPAT1
BnAnPRT
PpAnPRT
FcAnPRT
CbAnPRT
: ----MAAAS-----IKLSSSAKPLAPAPV----------------SHLLPLRAHHARPLPA--------------------RLPP : 40
: ----MAAAS-----IKLSSSAKPLAPAPV----------------SHLLPLRAHHARPLPA--------------------RLPP : 40
: ----MATAS-----LKLCS-PKPLAPGAP----------------SPLLR-RPSTARPVAR--------------------RLPP : 38
: ----MAAAS-----LKFAS-PKPLASAP-----------------SALLCGRRAQARPLPAS-------------------RLPP : 39
: MVIAVATTSSIVSGIKLSGILTSFNAVDDASSSCGRSNLTGVRIFPTLSRRRFSSIGAVSPIRGDAQSSFSRSSFACSQNLGLSG : 85
: ----MAVPTSIVSGIKLSGIVTSFNTPADVSSP-GR---ISVKVYPALPRRRFSSIGAVSSIRGDS----SRISFAYRQNLGLSG : 73
: ----MAAALASQRTWLPSSSVGPFRGSFAT--------------APASSPRVLGVPRKFAVE-------------------SGKW : 48
: ------------------------------------------------------------------------------------- : -
: ------------------------------------------------------------------------------------- : -
OsPAT1.1
OsPAT1.2
TaAnPRT
ZMAnPRT
AtPAT1
BnAnPRT
PpAnPRT
FcAnPRT
CbAnPRT
: PPRVAVQHTAARASDASPRTASFDKVLETLIGG-DHFSEEEAEATLRLLLE-EENEARIAAFLVLLRAKGETYEEIVGLAKAMIG : 123
: PPRVAVQHTAARASDASPRTASFDKVLETLIGG-DHFSEEEAEATLRLLLE-EENEARIAAFLVLLRAKGETYEEIVGLAKAMIG : 123
: -ARVAVQPAAAPAPAASPGTATFEQVLEQLIGG-ADFTEDEAEATLQLLLN-EKNEARIAAFLVLLRAKGETYDEIVGLAKAMMR : 120
: PTRVAVQPPAAP---VATRIGSFDKVLEALIGG-TDFSEEDAEATLKLLLD-EKDEARISAFLVLLRAKGETFEEIVGLAKAMLS : 119
: GFSAAEALPPACANASPSSIKSFNQLIETLIDR-VDLSETEAESSLEFLLN-EANEALISAFLVLLRAKGETYEEIVGLARAMMK : 168
: GLGAPEALPSVRENASASSIKSFNQLIESLIDR-VDLSESEAESSLEFLLN-DTNEALISAFLVLLRAKGETYEEIVGLARAMMK : 156
: EVLVRNVRAQSSTVTVPARTLSIPQVLEQLIQG-TNLTQAQTEEVMETLLA-DANPAIIGAFLALLRAKGETLDEVTGLASAMLS : 131
: --------------MSSAKSASLKPYIETLIDGKRHLSVEETKEAFSAILK-GADEVQVGSFLTLLRARGENPDEIAGAVEAMND : 70
: ---------------------MIKEAIVTLVSG-RSLATDEAAKVMEEIMEGEATPAQFGAFVTALRLKGETVDEIVGLSRTMRA : 63
: CCVRVDG-LDDAVDIVGTGGDGADTVNISTGSTILAAAAGAKVAKQGSRASSSACGSADVLEAFGVNIELGPEGIKRCVNEVGVG : 207
: CCVRVDG-LDDAVDIVGTGGDGADTVNISTGSTILAAAAGAKVAKQGSRASSSACGSADVLEAFGVNIELGPEGIKRCVNEVGVG : 207
: NCVLVEG-LHDAVDIVGTGGDGADTVNISTGSTILAAAAGAKVAKQGSRASSSACGSADVLEALGVNIELGPEGIKRCVNEVGVG : 204
: CCIRVDG-LDDAVDIVGTGGDGADTVNISTGSTILAAAAGAKVAKQGSRASSSACGSADVLEALGVNIELGPEGIKRCVNEVGVG : 203
: HARKVEG-LVDAVDIVGTGGDGANTVNISTGSSILAAACGAKVAKQGNRSSSSACGSADVLEALGVVLDLGPEGIKRCVEEGGIG : 252
: HARKVEG-LADAVDIVGTGGDGANTVNISTGSSILAAACGAKVAKQGSRSSSSACGSADVLEALGVVIDLGPEGIKRCVEESGIG : 240
: RAIPVRT-VPGSLDIVGTGGDGANTVNISTGSCILAAACGAKVAKHGSRSSSSACGSADVLETLGVAIDLGPEGVAKCVEDVGVG : 215
: ACNSVDLGDMKLLDIVGTGGDGADTINISTASTVLSAACGCIVAKAGNRSVSSACGSADVLEALGVKVDLTPSQVIECVNSVGIA : 155
: KAIPVST-DGLVVDTCGTGGDGSHTFNISTAAAFVAAGAGLKIAKHGNRAMSSQCGSADVLEALGVKIDLNAEQVQRCLKEIGIG : 147
OsPAT1.1
OsPAT1.2
TaAnPRT
ZMAnPRT
AtPAT1
BnAnPRT
PpAnPRT
FcAnPRT
CbAnPRT
OsPAT1.1
OsPAT1.2
TaAnPRT
ZMAnPRT
AtPAT1
BnAnPRT
PpAnPRT
FcAnPRT
CbAnPRT
: FMMSANYHPAMKIVKPVRKKLKIKTVFNILGPLLNPARVPYAVIGVYHENIVTKMAKAAQKFG-MKRALVVHS-KGLDEISPLGP : 290
: FMMSANYHPAMKIVKPVRKKLKIKTVFNILGPLLNPARVPYAVIGVYHENIVTKMAKAAQKFG-MKRALVVHS-KGLDEISPLGP : 290
: FMMSANYHPAMKIVRPVRKKLKIKTVFNILGPLLNPARVPYAVIGVYHENIVSKMAKAAQKFG-MQRALVVHS-KGLDEISPLGP : 287
: FMMSANYHPAMKIVRPVRKKLKIKTVFNILGPLLNPARVPHAVIGVYHENIVTKMAKAAQKFG-MKRALVVHS-KGLDEISPLGP : 286
: FMMSPMYHPAMKIVGPVRKKLKIKTVFNILGPMLNPARVSYAVVGVYHKDLVVKMAKALQRFG-MKRALVVHS-CGLDEMSPLGG : 335
: FMMAPMYHPATKIVSPVRKKLKIKTVFNVLGPMLNPARVSYAVVGVYHKNLVMKMAKALQLFG-MKRALVVHS-YGLDEMSPLGG : 323
: FMFAPTYHPAMKVVAPVRRSLKIKTAFNILGPMLNPSRAPHSIVGVYHDNLVEKMAKILQHFG-TKRTLVVHC-HGLDEMSPLGG : 298
: FMFAPVNHPSMKYVAPVRSKLGVRTVFNILGPMTNAASAQRAVIGVFHPELMELMAGALKKVGRVDHAVIIHG-IGLDEISPLGP : 239
: FMFAPVFHPAMKYAAAPRREIGIRTVFNILGPLTNPAGAKAQVLGVGDESLIEKLALVLQGLG-CQHALLVHGEDGLDEITITGK : 231
OsPAT1.1
OsPAT1.2
TaAnPRT
ZMAnPRT
AtPAT1
BnAnPRT
PpAnPRT
FcAnPRT
CbAnPRT
: GYILDVT----------PRKIEKMLFDPLDFGIPRCTLEDLKGGDPAFNAKVLQDVLAG--QRGSIADALVLNAAASLLVSGKVN : 363
: GYILDVT----------PRKIEKMLFDPLDFGIPRCTLEDLKGGDPAFNAKVLQDVLAG--QRGSIADALVLNAAASLLVSGKVN : 363
: GYILDVT----------PGKIEKMLFDPLDFGIPRCTLLDLKGGDPAFNAKVLQDVLAG--QRGSIADALVLNAAASLLVSGKVK : 360
: GYILDVT----------PEKIEKMFFDPLDFGIPRCTLEDLKGGDPAFNAKVLQDVLAG--QRGAIADALVLNAAASLLVSGKVK : 359
: GLVYDVT----------PEKIEEFSFDPLDFGIPRCTLEDLRGGGPDYNADVLRRVLSG--ESGAIADSLILNAAAALLVSNRVQ : 408
: GLVYDVT----------PEKIEEFSFDPLDFGIPRCTLEDLRGGGPDYNADVLRRVLSG--EKGSIADSLILNAAAALLVSNRVQ : 396
: GRVLEVT----------QDKIESFVFDPLNFGIPRCTLEDLKGGNADFNAKSLRNVFAG--EPGPIADSLILNAAAGIMACGLVK : 371
: ATLLEIKNIAADGVLPKVYETKTFEFDPLDIGIPRCELIDLKGGDPKENAEKFVDVLKGGDHTDAKRDSIVLNAGVGCWIYGLSN : 324
: THVCELK----------NDHIERYIVSPEDFGLPRASSGSIKGGDVKANAELLRNTISG--TLGSQRDVVLMNAAAALVAGDKVE : 304
| 440 460 |
| --- |
OsPAT1.1
OsPAT1.2
TaAnPRT
ZMAnPRT
AtPAT1
BnAnPRT
PpAnPRT
FcAnPRT
CbAnPRT
: TLHDGVALAQETQRSGEAINTLESWIKISNSC--------- : 395
: TLHDGVALAQETQRSGEAINTLESWIKISNVSTSDN----- : 399
: TLQEGVALAQETQRSGVAINTLESWIKVSNSC--------- : 392
: NLHDGVALAQETQRSGKAINTLESWIKKSNSSRD------- : 393
: TLAEGVTVAREVQSSGKAIKTLDSWINISNLAQKSQ----- : 444
: TLGEGVTLAREVHSSGKAIKTLDSWIHSSNLAQKSQ----- : 432
: DLGEGVSLARDVLRSGRANTVLDNWISLSQKLKAEEVSE-- : 410
: SIEDGCKLARTTLESGKASDLLQKWITVSQEIASKELNKPE : 365
: TLQQGVAMAKEVIDNGSAAAKLDQLIEFSRSLG-------- : 337
Figure S7. Alignment of OsPAT1 and some of its homologs. The reported conserved motifs were indicated with red boxes.

## Slide 8
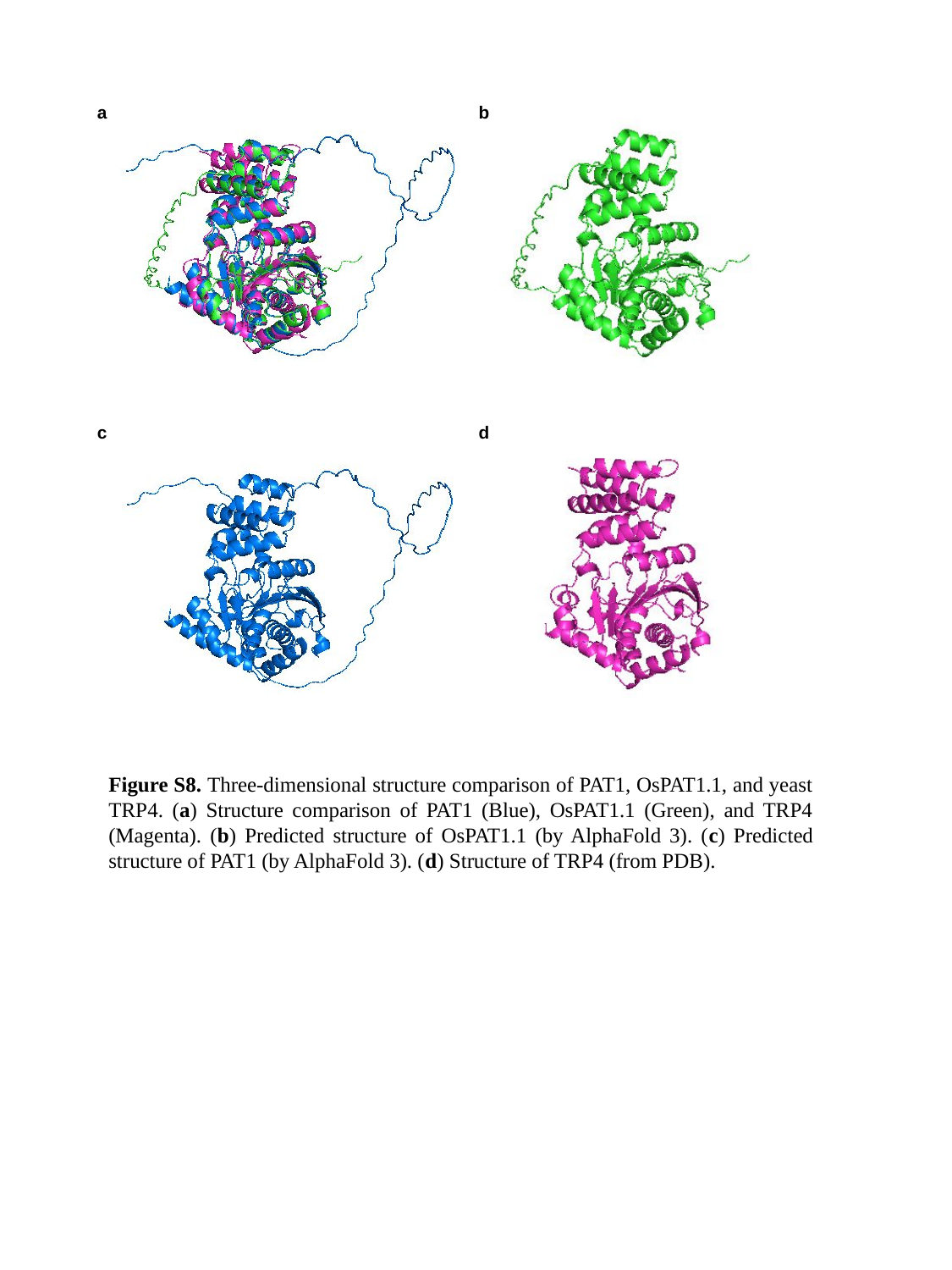

a
b
c
d
Figure S8. Three-dimensional structure comparison of PAT1, OsPAT1.1, and yeast TRP4. (a) Structure comparison of PAT1 (Blue), OsPAT1.1 (Green), and TRP4 (Magenta). (b) Predicted structure of OsPAT1.1 (by AlphaFold 3). (c) Predicted structure of PAT1 (by AlphaFold 3). (d) Structure of TRP4 (from PDB).

## Slide 9
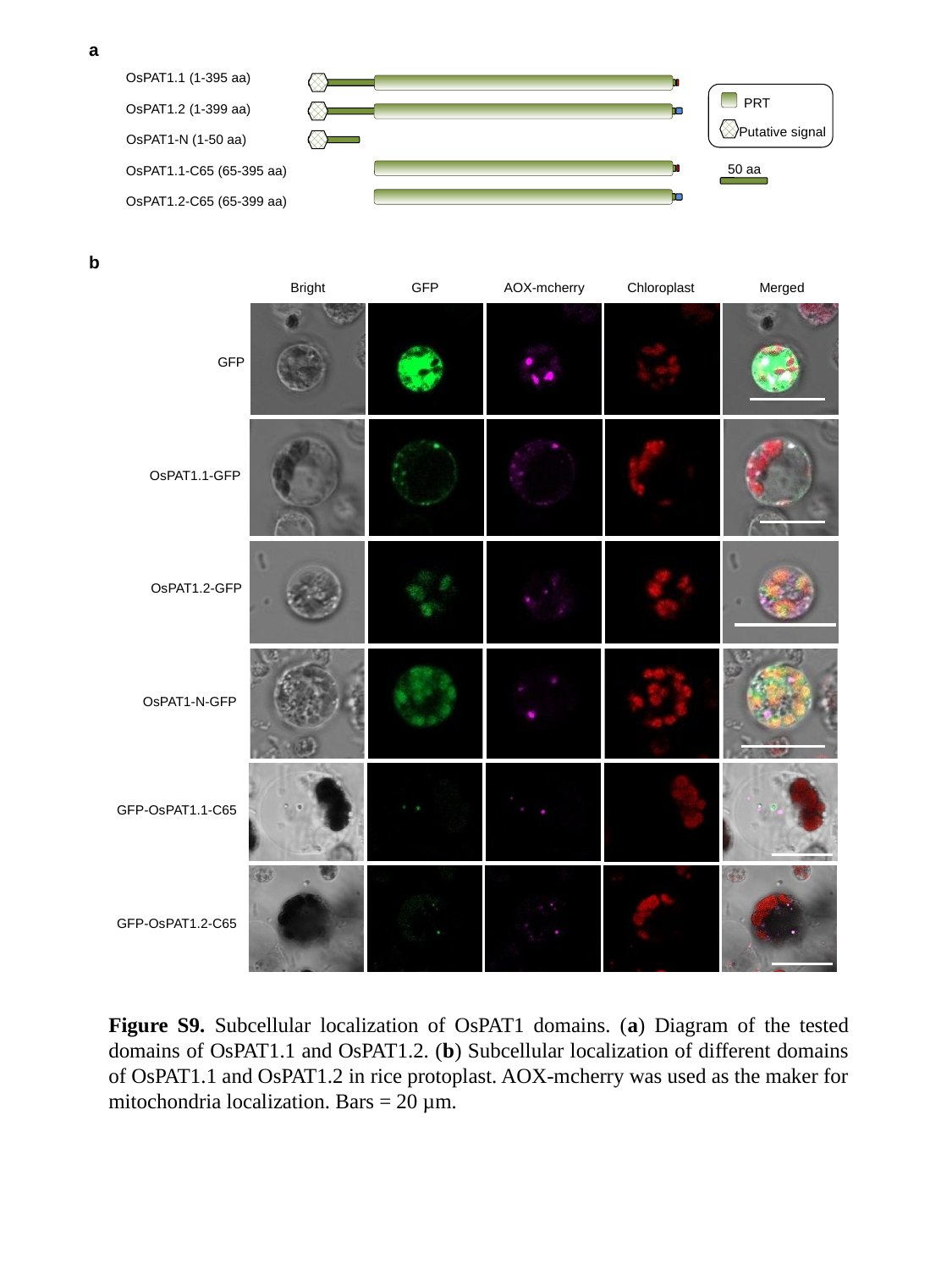

a
OsPAT1.1 (1-395 aa)
PRT
Putative signal
50 aa
OsPAT1.2 (1-399 aa)
OsPAT1-N (1-50 aa)
OsPAT1.1-C65 (65-395 aa)
OsPAT1.2-C65 (65-399 aa)
b
Bright
GFP
AOX-mcherry
Chloroplast
Merged
GFP
OsPAT1.1-GFP
OsPAT1.2-GFP
OsPAT1-N-GFP
GFP-OsPAT1.1-C65
GFP-OsPAT1.2-C65
Figure S9. Subcellular localization of OsPAT1 domains. (a) Diagram of the tested domains of OsPAT1.1 and OsPAT1.2. (b) Subcellular localization of different domains of OsPAT1.1 and OsPAT1.2 in rice protoplast. AOX-mcherry was used as the maker for mitochondria localization. Bars = 20 µm.

## Slide 10
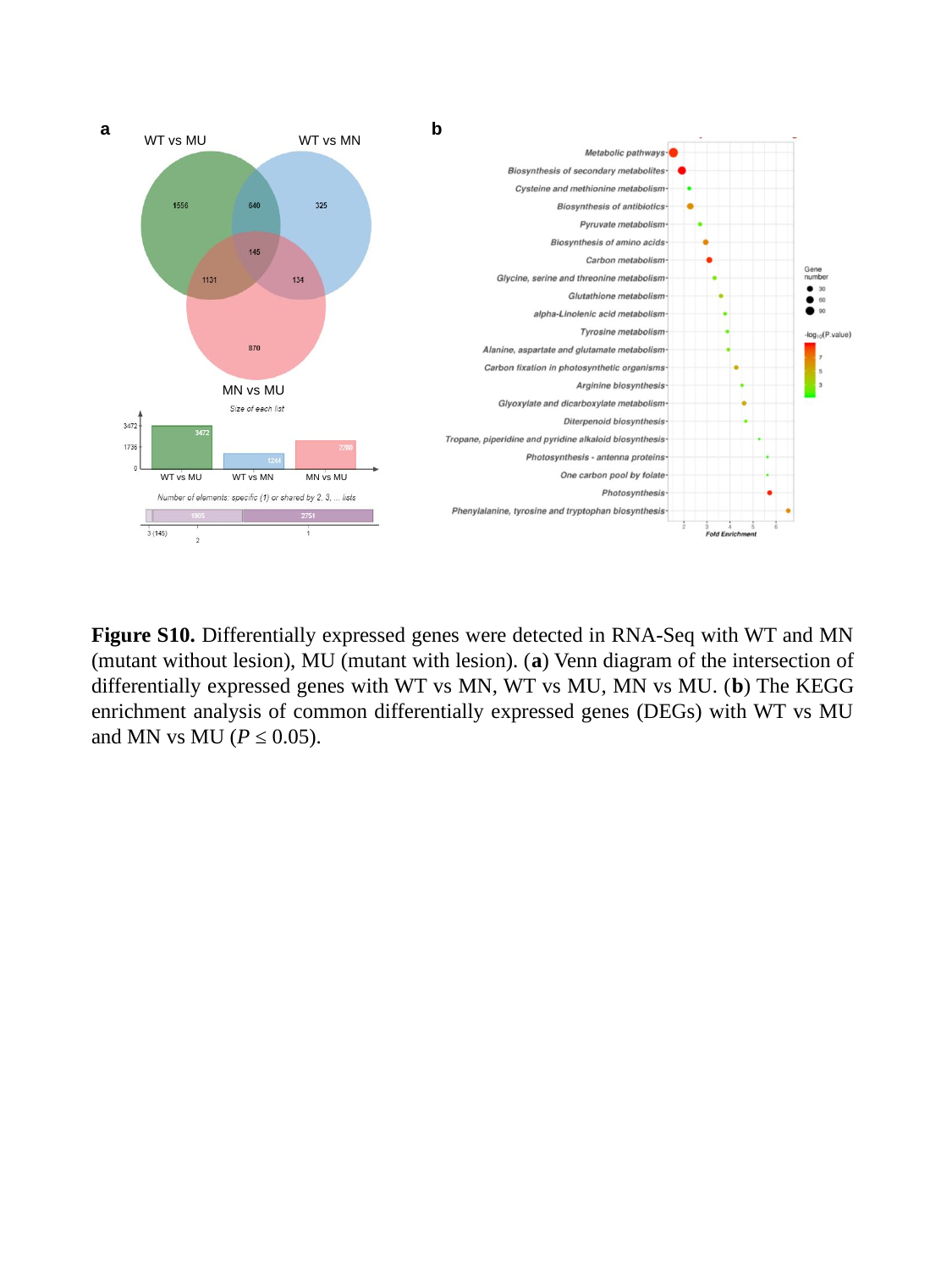

a
b
WT vs MU
WT vs MN
MN vs MU
MN vs MU
WT vs MU
WT vs MN
Figure S10. Differentially expressed genes were detected in RNA-Seq with WT and MN (mutant without lesion), MU (mutant with lesion). (a) Venn diagram of the intersection of differentially expressed genes with WT vs MN, WT vs MU, MN vs MU. (b) The KEGG enrichment analysis of common differentially expressed genes (DEGs) with WT vs MU and MN vs MU (P ≤ 0.05).

## Slide 11
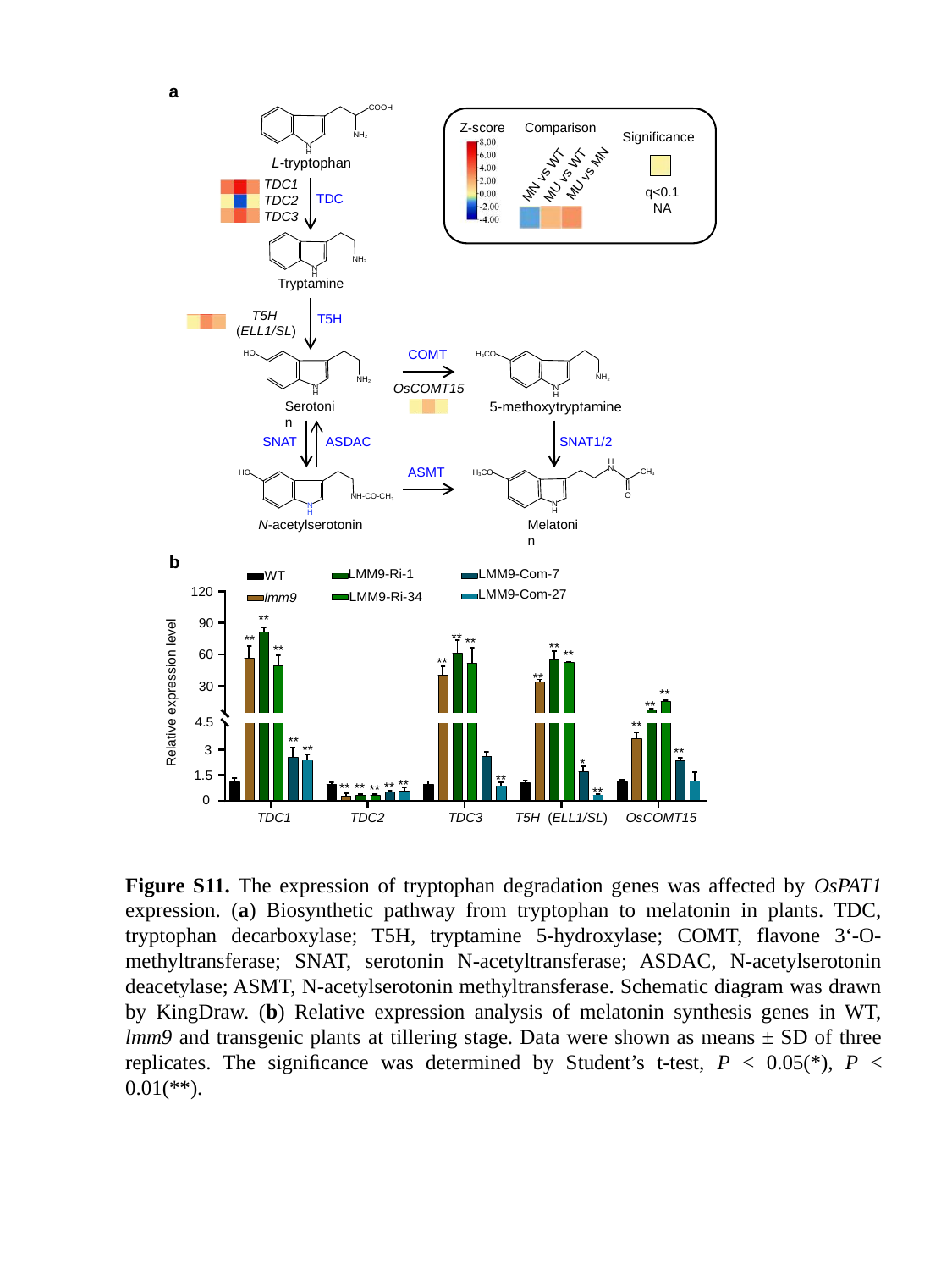

a
Z-score
Comparison
Significance
L-tryptophan
MU vs MN
MN vs WT
MU vs WT
TDC1
TDC2
TDC3
q<0.1
NA
TDC
Tryptamine
T5H
(ELL1/SL)
T5H
COMT
OsCOMT15
5-methoxytryptamine
Serotonin
SNAT
ASDAC
SNAT1/2
ASMT
N-acetylserotonin
Melatonin
b
LMM9-Ri-1
LMM9-Com-7
WT
LMM9-Com-27
LMM9-Ri-34
lmm9
120
**
90
Relative expression level
**
**
**
**
**
60
**
**
**
30
**
**
4.5
**
**
3
**
**
*
1.5
**
**
**
**
**
**
**
0
T5H (ELL1/SL)
TDC1
TDC2
TDC3
OsCOMT15
Figure S11. The expression of tryptophan degradation genes was affected by OsPAT1 expression. (a) Biosynthetic pathway from tryptophan to melatonin in plants. TDC, tryptophan decarboxylase; T5H, tryptamine 5-hydroxylase; COMT, flavone 3‘-O-methyltransferase; SNAT, serotonin N-acetyltransferase; ASDAC, N-acetylserotonin deacetylase; ASMT, N‐acetylserotonin methyltransferase. Schematic diagram was drawn by KingDraw. (b) Relative expression analysis of melatonin synthesis genes in WT, lmm9 and transgenic plants at tillering stage. Data were shown as means ± SD of three replicates. The signiﬁcance was determined by Student’s t-test, P < 0.05(*), P < 0.01(**).
